# Design, Synthesis, and Repurposing of Rosmarinic Acid-β-Amino-α-Ketoamide Hybrids as Antileishmanial Agents

**DOI:** 10.3390/ph16111594

**Published:** 2023-11-12

**Authors:** Ahmed H.E. Hassan, Waleed A. Bayoumi, Selwan M. El-Sayed, Trong-Nhat Phan, Taegeun Oh, Gyeongpyo Ham, Kazem Mahmoud, Joo Hwan No, Yong Sup Lee

**Affiliations:** 1 Department of Medicinal Chemistry, Faculty of Pharmacy, Mansoura University, Mansoura 35516, Egypt; 2 Medicinal Chemistry Laboratory, Department of Pharmacy, College of Pharmacy, Kyung Hee University, Seoul 02447, Republic of Korea; 3 Department of Pharmaceutical Organic Chemistry, Faculty of Pharmacy, Mansoura University, Mansoura 35516, Egypt; 4 Institute of Applied Science and Technology, School of Technology, Van Lang University, Ho Chi Minh City 700000, Vietnam; 5 Faculty of Applied Technology, School of Technology, Van Lang University, Ho Chi Minh City 700000, Vietnam; 6 Department of Fundamental Pharmaceutical Sciences, Kyung Hee University, Seoul 02447, Republic of Korea; 7 Department of Pharmaceutical Chemistry, Faculty of Pharmacy, Egyptian Russian University, Badr City 11829, Egypt; 8 Host-Parasite Research Laboratory, Institut Pasteur Korea, Seongnam-si 13488, Gyeonggi-do, Republic of Korea

**Keywords:** antileishmanial agents, visceral leishmaniasis, *Leishmania donovani*, promastigotes, cinnamoyl-β-amino-α-ketoamides, drug repurposing

## Abstract

A series of rosmarinic acid-β-amino-α-ketoamide hybrids were synthesized and rationally repurposed towards the identification of new antileishmanial hit compounds. Two hybrids, **2g** and **2h,** showed promising activity (IC_50_ values of 9.5 and 8.8 μM against *Leishmania donovani* promastigotes, respectively). Their activities were comparable to erufosine. In addition, cytotoxicity evaluation employing human THP-1 cells revealed that the two hybrids **2g** and **2h** possess no cytotoxic effects up to 100 µM, while erufosine possessed cytotoxicity with CC_50_ value of 19.4 µM. In silico docking provided insights into structure–activity relationship emphasizing the importance of the aliphatic chain at the α-carbon of the cinnamoyl carbonyl group establishing favorable binding interactions with *Ld*CALP and *L*ARG in both hybrids **2g** and **2h**. In light of these findings, hybrids **2g** and **2h** are suggested as potential safe antileishmanial hit compounds for further development of anti-leishmanial agents.

## 1. Introduction

Despite their negative impacts on health, society, and economy, development of new therapies for neglected tropical diseases (NTDs) is still receiving little attention and low priority by pharmaceutical drug development entities to the combating of NTDs [1]. This might be attributed to the fact that the most affected communities are the poorest populations of underdeveloped countries of Africa, Asia, and Latin America. Amongst NTDs, leishmaniasis is a major concern. It is reported that 12 million patients are currently afflicted by leishmaniasis [2]. The disease could exist in one of three main clinical manifestations, cutaneous leishmaniasis (CL), mucocutaneous leishmaniasis (MCL), or visceral leishmaniasis (VL) [3]. The latter clinical form is a fatal NTD if left untreated [4,5]. The global incidence of VL is estimated at 0.2–0.4 million cases [2]. Unfortunately, current available VL therapies involve the use of drugs associated with severe toxic/side effects, such as liposomal amphotericin B, miltefosine, or pentavalent antimonial salts [6,7,8,9,10]. Except for miltefosine, the current few available treatment options require invasive parenteral administration route and, in addition, refrigerated transport and storage are needed which impose logistics challenges and costs. Meanwhile, the oral drug miltefosine is teratogenic and has significant toxicities. Undoubtedly, there is a pressing need for the development of new treatments options for VL [11,12,13].

Natural products play an important role in drug discovery and development [14,15,16,17]. Rather than serving as real drugs, the majority constitute a rich reservoir of privileged bioactive scaffolds which could be exploited as starting points to develop novel bioactive compounds. In this lieu, the hybridization of natural products’ fragments with other fragments from natural or synthetic origin can afford potential bioactive compounds [18,19,20]. In principle, hybridization is a drug design strategy that extends beyond natural products and proved fruitful in achieving potential bioactive multifunctional molecules [20,21,22,23,24,25,26,27].

Repurposing (also known as repositioning or reprofiling) is a useful approach that makes use of already known chemical entities through repurposing them to new therapeutic applications. Such repurposed chemical entities could be already approved, withdrawn, shelved, investigational drugs, or even failed and development-stalled molecules [28,29]. A major benefit from adopting a repurposing strategy is that it enables expeditious discovery process through reduction of initial discovery phase time. In addition, repurposing can help dramatically cut down the costs which is a major concern in developing therapeutics for rare and neglected disease including leishmaniasis. In fact, several antileishmanial drugs are repurposed molecules such as amphotericin B and miltefosine. As for the pressing need for novel antileishmanial agents, particularly for the fatal visceral leishmaniasis, the current effort was conducted adopting natural products-based strategy based on hybridization and coupled with repurposing strategies.

## 2. Results and Discussion

### 2.1. Design and Repurposing Rational

**Figure 1:**
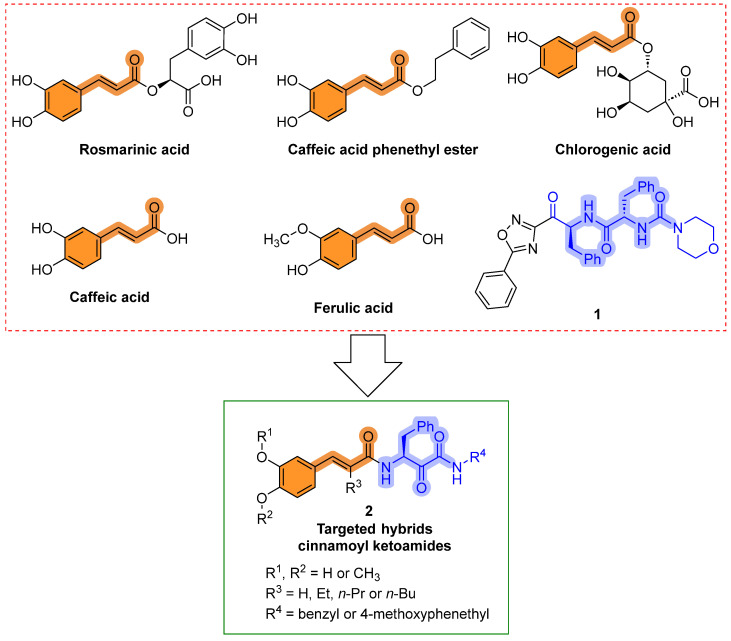
Design rational of targeted anticipated antileishmanial compounds **2** as hybrids of antileishmanial natural products incorporating cinnamoyl fragment and calpain inhibitors agents incorporating β-amino-α-ketoamide-based small peptide moiety.

Rosmarinic acid, caffeic acid phenethyl ester (CAPE) and chlorogenic acid (Figure 1) are natural esters of caffeic acid, which is a natural 3,4-dihydroxycinnamic acid derivative. Interestingly, rosmarinic, CAPE, and chlorogenic acids were found to exhibit antileishmanial activity. Thus, rosmarinic was reported to possess IC_50_ values of 61.0 and 57.3 µM against promastigotes of *L. amazonensis* and *L. infantum*, respectively [30,31]. In addition, CAPE possessed IC_50_ value of 51.0 μg/mL against promastigotes of *L. infantum* [32]. Meanwhile, chlorogenic showed a poor IC_50_ value of >500.0 µM against promastigotes of *L. amazonensis* [30]. In fact, the natural products, caffeic acid itself and its 3-methylated analog, ferulic acid, possess antileishmanial activity suggesting 3,4-dioxygenated cinnamic acid moiety as a privileged antileishmanial fragment [30,31,33]. Interestingly, rosmarinic, chlorogenic, and caffeic acids were reported to inhibit *L*. *amazonensis* arginase (*La*ARG) and *L. infantum* arginase (*Li*ARG), a potential target involved in parasite growth and infectivity [30,31]. It has been reported that the hydroxyl groups of 3,4-positions have important roles in binding *La*ARG [30].

Cysteine proteases play axial roles in proliferation and pathogenicity of *Leishmania* [34]. Hence, some small peptide-based cysteine protease inhibitors were discovered to possess antileishmanial activity. Thus, derivative **1** (Figure 1) was also reported to possess potential antileishmanial activity against *L. mexicana* [35]. In addition, several calpain inhibitors, such as MDL28170, were repurposed as antileishmanial agents showing significant antileishmanial activity against *L. amazonensis* promastigotes [36,37,38]. In fact, *Leishmania* possesses calpain-like cysteine protease (*L*CALP), which is a potential target for development of antileishmanial agents [39]. The structure of derivative **1** involves an aromatic system coupled to the small peptide moiety which is responsible for inhibition of cysteine proteases.

Aiming to develop novel antileishmanial agents impacting multiple antileishmanial biological targets rather than a single target, a hybrid structure was designed. The designed hybrid structure (**2**, Figure 1) possessed a 3,4-dioxygenated-cinnamoyl fragment as a privileged *L*ARG inhibiting moiety coupled with a β-amino-α-ketoamide-based small peptide fragment as a privileged *L*CALP inhibiting moiety small peptide moiety. Thus, the 3,4-dioxygenated-cinnamoyl fragment replaced the aromatic systems of derivative **1**. The dioxygenation pattern of the cinnamoyl moiety was maintained at 3,4-position, as such pattern was reported to be required for *L*ARG inhibition. The 3,4-dioxygenated substituents were planned as hydroxy and/or methoxy groups. While the α-carbon was unsubstituted in the above-mentioned cinnamoyl natural products, α-unsubstituted cinnamoyl moieties as well as α-alkyl cinnamoyl moieties were planned to be explored to interrogate the steric and lipophilic effects at this position on the activity. In addition, to investigate the impact of the C-terminal amide substituent of the β-amino-α-ketoamide-based peptide fragment on the activity, it was varied between *N*-benzyl and the longer *N*-(4-methoxyphenethyl) which also possessed a polar methoxy group. In the literature, some compounds having a structure conforming to the targeted design were reported as antioxidants and human calpain inhibitors [40]. Hence, this effort might be considered as repurposing of these compounds towards discovery of new antileishmanial hit compounds.

### 2.2. Chemistry

**Scheme 1:**
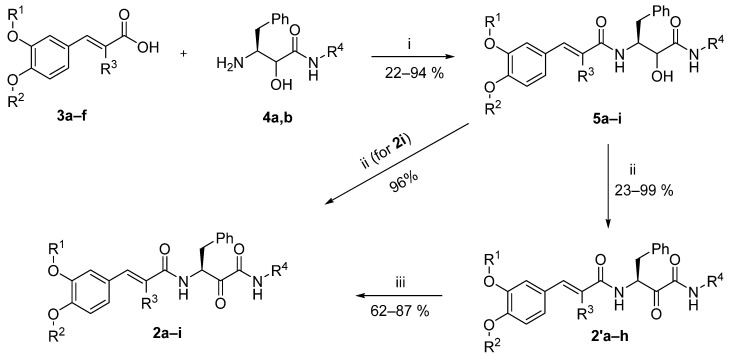
Synthesis of target cinnamoyl-β-amino-α-ketoamide hybrids (**2a–i**). Reagents and conditions: (i) EDC, HOBt, DMF, stir 0 °C–rt; (ii) Dess-Martin periodinane, DMF, 0 °C–rt, 10% Na_2_S_2_O_3_ solution; (iii) 1% methanolic HCl, 70 °C.

The synthesis of the targeted hybrids **2a–i** was accomplished in two or three linear steps (Scheme 1). First, coupling of the appropriate MOM-protected and/or *O*-methylated 3,4-dihydroxycinnamic acid derivative **3a–f** with the appropriate β-amino-α-hydroxyamide **4a** or **4b** was achieved using 1-ethyl-3-(3-dimethylaminopropyl)carbodiimide. HCl (EDC) and 1-hydroxybenzotriazole (HOBt) at an ambient temperature to afford the corresponding cinnamoyl-β-amino-α-hydroxyamides **5a–i** [40]. Using the mild Dess–Martin periodinane oxidation conditions, cinnamoyl-β-amino-α-hydroxyamides **5a–i** were converted to MOM-protected cinnamoyl-β-amino-α-ketoamides **2′a–h** or 3,4-dimethoxycinamoyl-β-amino-α-ketoamide **2i** [40]. Finally, target 3,4-dioxygenated-cinamoyl-β-amino-α-ketoamides **2a–i** having hydroxy substituents were obtained from intermediates **2′a–h** after deprotection of MOM with 1% methanolic HCl.

### 2.3. Biological Evaluations

#### 2.3.1. Evaluation of Antileishmanial Activity

##### In Vitro *L. donovani* Promastigotes-Based Evaluation Model

Using an in vitro promastigotes-based model, the activity of the synthesized target compounds **2a–i** was assessed against the promastigote stage of *L. donovani*. A double-concentration assay was performed for each compound at 50 and 25 µM concentrations adopting a resazurin-based assay system [41]. Erufosine was used as a positive reference standard drug. The results are summarized in Table 1.

By examination of the results, it was observed that most of the target cinnamoyl-β-amino-α-ketoamides hybrids (**2a–i**) showed promising results comparable to the standard drugs. At 50 µM and 25 µM concentrations, the range of % inhibition was (100–104) and (98–107), respectively. Only hybrid **2b**, having unsubstituted α-carbon of the cinnamoyl moiety coupled with *N*-4-methoxyphenethyl substituent at the peptide’s C-terminal, showed deteriorated activity at the lower 25 µM concentration. However, corresponding hybrids **2e** and **2g,** which differ only from hybrid **2b** by having a *n*-propyl or *n*-butyl substituent at α-carbon of the cinnamoyl moiety, maintained high activity at both tested concentrations. This might suggest that the presence of an alkyl substituent at this position is beneficial for the activity. The results showed also that hybrid **2i** having a cinnamoyl moiety featuring dimethoxy substitution pattern at the phenyl ring and *n*-butyl substituent at the α-carbon coupled with cinnamoyl part *N*-4-methoxyphenethyl substituent at the peptide’s C-terminal showed reduced activity at the lower 25 µM concentration. This might suggest that at least one hydroxy substituent at the phenyl ring of the cinnamoyl moiety is required for maintaining good activity.

##### In Vitro Potency Evaluation against *L. donovani* Promastigotes

Among the evaluated compounds, seven derivatives maintained a high inhibitory activity at 25 and 50 μM concentrations which was similar to erufosine. Therefore, they were advanced to assessment of potency to evaluate their quality as antileishmanial hit compounds. The less potent compounds would be distinguished from the more potent ones by the lower concentrations used to generate the dose–response curve, allowing for more rigorous evaluation of the potency of these compounds that exhibited ≥100 inhibition at both 50 and 25 μM concentrations in the previous double concentrations assay. Erufosine was used as positive controls for comparison. The results of determined IC_50_ values for the growth of *L. donovani* are summarized in Table 2.

Comparing the determined IC_50_ values for compounds **2a**, **2c**, **2d** and **2f** that differ only by having variable alkyl chains length at α-carbon of the cinnamoyl moiety while sharing the same *N*-benzyl substituent at the peptide’s C-terminal and 3,4-dihydroxy substituents at the phenyl of the cinnamoyl moiety indicated an influential role for α-carbon substituent. Thus, derivative **2f** having the longer *n*-butyl chain as a substituent at α-carbon of the cinnamoyl moiety was the most potent among these four compounds while potency gradually decreased in proportional correlation to the decrease of alkyl substituent length at α-carbon. Comparing the potency of compounds **2d** and **2f** versus compounds **2e** and **2g,** respectively, indicates that compounds having *N*-4-methoxyphenethyl substituent at the peptide’s C-terminal are more potent relative to corresponding derivatives possessing *N*-benzyl substituent. Finally, comparing the activity of compound **2g** versus compound **2h** shows that the good potency of compound **2g** is slightly enhanced upon converting the 3-hydroxy substituent at the phenyl of the cinnamoyl moiety into 3-methoxy, while maintaining the 4-hydroxy substituent to afford compound **2h**. Overall, hybrids **2g** and **2h** were identified as promising compounds eliciting comparable potencies to the reference drug erufosine (IC_50_ values of 9.5 and 8.8 μM versus 9.8 μM for compounds **2g**, **2h**, and erufosine, respectively). In summary, the deduced SAR might be: (1) longer *n*-butyl substituent at cinnamoyl α-carbon is better than smaller alkyl chains; (2) *N*-4-methoxyphenethyl substituent at the peptide’s C-terminal is more preferred than *N*-benzyl; and (3) 3-methoxy-4-hydroxy substituents at the phenyl of the cinnamoyl moiety might be preferred than 3,4-dihydroxy substituents.

##### Structure–Activity Relationship

**Figure 2:**
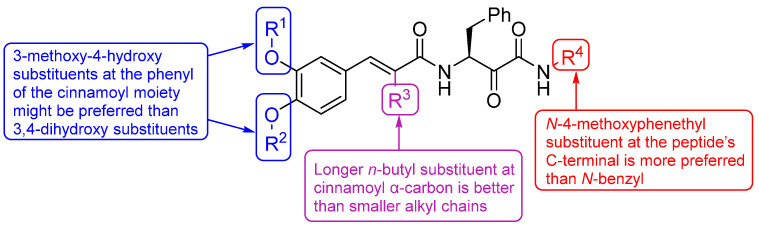
Structure–activity relationship of cinnamoyl-β-amino-α-ketoamides hybrids.

As could be deduced from the determined IC_50_ values against *L. donovani* promastigotes, SAR might be summarized as follows: (1) longer *n*-butyl substituent at cinnamoyl α-carbon is better than smaller alkyl chains; (2) *N*-4-methoxyphenethyl substituent at the peptide’s C-terminal is more preferred than *N*-benzyl; and (3) 3-methoxy-4-hydroxy substituents at the phenyl of the cinnamoyl moiety might be preferred than 3,4-dihydroxy substituents (Figure 2).

##### In Vitro Safety Evaluation

As for the considerable toxicities of current antileishmanial therapies, a promising drug candidate should be selective eliciting no potential cytotoxicity towards human cells. To evaluate the quality of identified most active antileishmanial cinnamoyl-β-amino-α-ketoamide hybrids **2g** and **2h**, a preliminary cytotoxicity was conducted using monocytic THP-1 human cell line. The newly introduced hit compound needs to be safe and non-toxic to human cells. A comparison with the standard drug erufosine was performed. In contrast to erufosine that showed CC_50_ of 19.4 μM, the tested two hybrids **2g** and **2h** showed interestingly no potential cytotoxicity with determined C_50_ values > 100 µM for both compounds (Table 3). In light of these findings, it might be inferred that both cinnamoyl ketoamide hybrids **2g** and **2h** could be selective antileishmanial hit compounds for future development.

### 2.4. Molecular Modeling Study

Molecular docking simulations are considered as a successful tool that helps get distinct insights into the binding modes of small molecules to their receptors. This might be applied in the early stages of hit discovery in order to provide insights for future lead optimization studies. The in vitro biological evaluation of the target compounds unveiled two hybrids **2g** and **2h** as the most potential compounds. Accordingly, molecular docking study was performed using hybrids **2g** and **2h** against both *Leishmania* arginase *L*ARG and *L. donovani* calpain-like protein (*Ld*CALP), using rosmarinic and the cysteine protease inhibitor **1**, respectively.

**Figure 3:**
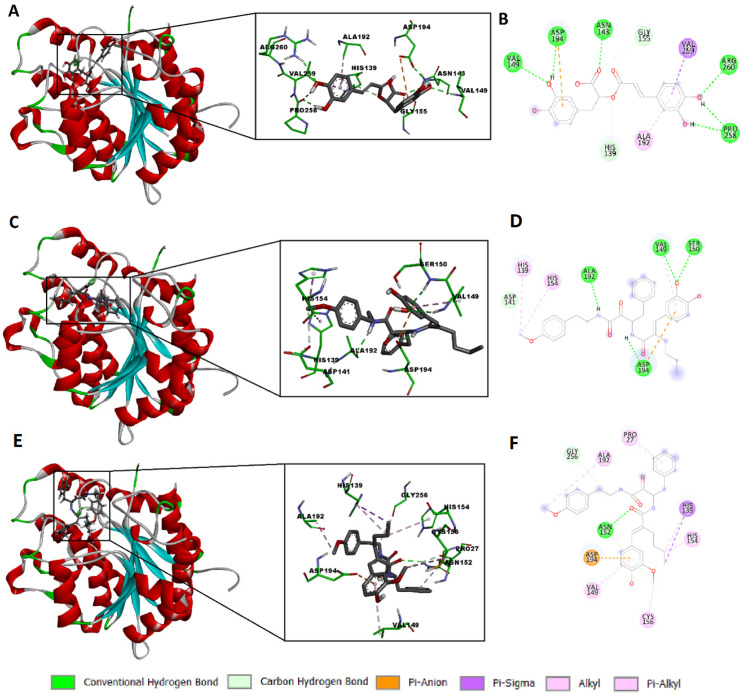
In silico calculated binding modes of the reference ligand rosmarinic acid and hybrids **2g** and **2h** with the catalytic binding site of arginase: (**A**) Calculated binding pose of the reference ligand rosmarinic acid (**7**) in 3D; (**B**) Calculated binding pose of the reference ligand rosmarinic acid (**7**) in 2D; (**C**) Calculated binding pose of **2g** in 3D; (**D**) Calculated binding pose of **2g** in 2D; (**E**) Calculated binding pose of **2h** in 3D; and (**F**) Calculated binding pose of **2h** in 2D.

First, the docking simulation was performed using the crystal structure of *L*ARG enzyme (PDB ID: 5HJA). Both hybrids **2g** and **2h** as well as the reference rosmarinic docked well inside the catalytic binding site of the receptor located within a β-sheets-rich domain between two α-helices-rich domains (Figure 3). The calculated binding scores of the reference rosmarinic acid and hybrids **2g** and **2h** were −6.24, −7.34, and −7.44 kcal/mol, respectively (Table 4). This suggested both hybrids **2g** and **2h** possessed at least equivalent or more potency as arginase inhibitors relative to rosmarinic acid. The network of binding interactions formed by the reference ligand included four hydrogen bonds between the hydroxyl substituents on its phenyl rings and Asp194, Val149, Pro258, and Arg260, in addition to another hydrogen bond between the carboxylic acid moiety and Asn143. Additionally, it was found that a π-anion interaction with Asp194 was formed via the phenyl ring of the benzoic acid functionality. Moreover, rosmarinic acid was found to form hydrophobic π-sigma and π-alkyl interactions between the phenyl ring of the cinnamoyl moiety and Val259, in addition to two carbon-hydrogen interactions with Gly155 and His139. Close to this pattern of interactions, **2g** showed two favorable hydrogen bonds between the phenolic hydroxyl substitution on the cinnamoyl moiety and Val149 and Ser150, in addition to other two hydrogen bonds between NH of both amide and β-ketoamide moieties and Ala192 and Asp194. A network of hydrophobic π-alkyl and carbon-hydrogen interactions with His139, His154, and Asp141 was formed with the methoxyphenyl moiety. Moreover, **2g** formed a π-anion interaction between the phenyl ring of the cinnamoyl moiety and Asp194. On the other side, **2h** was able to form hydrogen bonds between the carbonyl group of the amide functionality and Asn152, π-anion interaction between the phenyl ring of the cinnamoyl moiety and Asp194. Additionally, the *n*-butyl aliphatic moiety of **2h** was involved in a π–sigma bond with His139, and favorable hydrophobic alkyl and π-alkyl bindings with His139, His154. Moreover, the network of various hydrophobic alkyl, π-alkyl, and carbon–hydrogen interactions were formed with Pro27, Cys156, Val149, Asn152, Ala192, and Gly256. These results suggested that cinnamoyl moiety represents a privileged moiety that was involved in different favorable binding interactions of **2g** and **2h** with the substrate binding site of (5HJA).

Another docking trial applying the *L. donovani* calpain-like protein (*Ld*CALP) as a target receptor was performed. Since no crystal structure of *Ld*CALP is identified, the homology model of the catalytic domain of *Ld*CALP was obtained from SWISS-MODEL (E9BTE3_LEIDB). The obtained model was utilized in the docking experiment and compound **1** (cysteine protease inhibitor) was applied as reference ligand for a comparative evaluation of binding affinity and interactions to *Ld*CALP.

**Figure 4:**
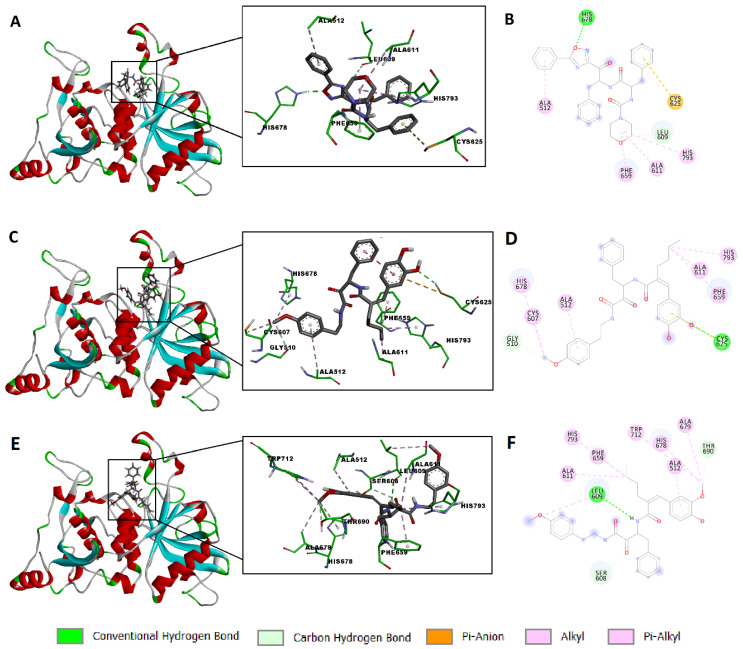
In silico calculated binding modes of the reference ligand (compound **1**) and compounds **2g** and **2h** with the substrate site of *Ld*CALP: (**A**) Calculated binding pose of the reference ligand MDL-28170 in 3D; (**B**) Calculated binding pose of the reference ligand MDL-28170 in 2D; (**C**) Calculated binding pose of **2g** in 3D; (**D**) Calculated binding pose of **2g** in 2D; (**E**) Calculated binding pose of **2h** in 3D; and (**F**) Calculated binding pose of **2h** in 2D.

The examination of the calculated binding energy scores of the investigated molecules illustrated that the reference ligand, **1**, possesses energy score of −8.41 Kcal/mol while **2g** and **2h** have comparable scores of −7.80 and −8.10 Kcal/mol, respectively (Table 4). Moreover, tested compounds (**2g** and **2h**) and the reference ligand were found to form a network of favorable binding interactions with the substrate site of *Ld*CALP, which is located in the middle cleft between the α-helices-rich domain and β-sheets-rich domain (Figure 4). The examination of the binding mode of the reference ligand showed that it formed a favorable hydrogen bond between its oxadiazole ring and His678 amino acid residue within the catalytic substrate site of *Ld*CALP. Also, it formed π–sulfur interaction between the phenethyl side chain and Cys625. The morpholine ring could establish hydrophobic alkyl, π–alkyl, and carbon–hydrogen interactions with Phe659, Ala611, His793, and Leu609 amino acid residues. Moreover, the terminal phenyl moiety formed π-alkyl bonding with Ala512. Interestingly, the straight *n*-butyl aliphatic chain at the α-carbon of the cinnamoyl carbonyl group within both **2g** and **2h** was involved in alkyl and π-alkyl hydrophobic interactions with Ala611, Phe659, and His793. These interactions were the same as those formed between the morpholine ring within the reference molecule and the same amino acid residues, indicating the crucial role of the *n*-butyl aliphatic chain in the *Ld*CALP inhibitory activity of the investigated compounds. In addition to these hydrophobic interactions, **2g** exhibited network of favorable bonds including hydrogen bond and π–sulfur interaction between the dihydroxyphenyl functionality and Cys625, in addition to alkyl, π–alkyl, and carbon–hydrogen interactions between the terminal methoxyphenyl moiety and Ala512, Cys607, His678, and Gly510. Moreover, **2h** was able to establish hydrogen bonding with Leu609, in addition to hydrophobic alkyl, π–alkyl, and carbon–hydrogen interactions between its 3-methoxy-4-hydroxyphenyl moiety and Ala679, Ala512, His678, Trp712, and Thr690. Another hydrophobic carbon-hydrogen bond was formed between the O-atom of the β-ketoamide moiety and Ser608, in addition to π–alkyl interaction between the terminal methoxyphenyl moiety and Leu609 (Figure 4).

The combined results of *L*ARG and *Ld*CALP docking experiment suggest that the privileged cinnamoyl moiety, as well as the straight aliphatic *n*-butyl chain at the α-carbon of the cinnamoyl carbonyl group within both **2g** and **2h**, can provide useful insights into the structural features required for possible inhibitory activity of the tested compounds against *Ld*CALP and arginase enzyme. These findings came in accordance with the SAR findings where the docking of **2g** and **2h** unveiled that the 3-methoxy group of the 3-methoxy-4-hydroxy substituents at the phenyl of the cinnamoyl moiety, *n*-butyl substituent at cinnamoyl α-carbon, and *N*-4-methoxyphenethyl substituent at the peptide’s C-terminal were involved in various interactions with both *L*ARG and *Ld*CALP.

## 3. Materials and Methods

### 3.1. Chemistry

Compounds were prepared as reported previously [40] and detailed in the Supplementary Information.

### 3.2. Biological Evaluations

#### Evaluations of Antileishmanial Activity and Safety Assessment

The adopted evaluation procedures were conducted following reported protocols (Supplementary Information) [42,43,44,45,46].

### 3.3. In Silico Study

The reported structures of *L. donovani* calpain-like cysteine peptidase (*Ld*CALP; SWISS-MODEL) [47] and *Leishmania* arginase (*L*ARG; PDB ID: 5HJA) were used for docking which was carried following standard protocols [47].

## 4. Conclusions

In this study, a logical drug repurposing rational of cinnamoyl-β-amino-α-ketoamides hybrids **2a–i** was addressed in pursuit of the discovery of antileishmanial hit compounds. Two compounds (**2g** and **2h**) were discovered as potential hits against leishmaniasis. The first step was to construct a rationally designed hybrid structure previously reported towards the development of calpain inhibitors. The synthesis of the target hybrids was achieved through concise and linear steps. The target hybrids were evaluated for their antileishmanial activity against *L. donovani*. The adopted molecular hybridization strategy succeeded in combining the significant calpain inhibitory activity of peptidyl fragment with the antileishmanial activity of natural cinnamic acid derivative moiety. Two potential hits, **2g** and **2h,** were identified with more potent activity than the standard erufosine. Preliminary safety assessment proved the high safety margin of **2g** and **2h** to human cells. In silico studies introduced the cinnamoyl moiety and the straight aliphatic *n*-butyl chain at the α-carbon of the cinnamoyl carbonyl group within both **2g** and **2h** as crucial scaffolds for the formation of favorable binding interactions within the substrate binding sites of *L*ARG and *Ld*CALP. While the current work was based on only in vitro and in silico investigations, its outcomes position cinnamoyl-ketoamide hybrids **2g** and **2h** as antileishmanial hit compounds favored for future research into possible antileishmanial agents. Future studies might be directed into more advanced in vitro and in vivo evaluations as well as optimization studies.

## Figures and Tables

**Table 1:** % Growth inhibition of *L. donovani* promastigotes elicited by cinnamoyl-β-amino-α-ketoamides hybrids **2a–i**.

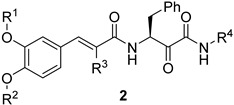						
Compound	R^1^	R^2^	R^3^	R^4^	% Inhibition at 50 µM ^1^	% Inhibition at 25 µM ^1^
**2a**	H	H	H	benzyl	103 ± 0.4	97.82 ± 1.0
**2b**	H	H	H	4-methoxyphenethyl	72.83 ± 0.4	44.02 ± 1.2
**2c**	H	H	ethyl	benzyl	101 ± 0.2	104 ± 0.3
**2d**	H	H	*n*-propyl	benzyl	102 ± 0.1	107 ± 0.4
**2e**	H	H	*n*-propyl	4-methoxyphenethyl	104 ± 0.1	104 ± 0.2
**2f**	H	H	*n*-butyl	benzyl	100 ± 0.3	104 ± 0.4
**2g**	H	H	*n*-butyl	4-methoxyphenethyl	103 ± 0.1	107 ± 0.1
**2h**	methyl	H	*n*-butyl	4-methoxyphenethyl	100 ± 0.3	103 ± 0.2
**2i**	methyl	methyl	*n*-butyl	4-methoxyphenethyl	94.16 ± 1.2	79.85 ± 1.8
**Erufosine**					107.6 ± 0.3	100 ± 1.0

^1^ % Inhibition of growth of *L. donovani* promastigotes after incubation with 50 and 25 µM concentrations for 3 days relative to control.

**Table 2:** Determined IC_50_ values against *L. donovani* promastigotes for the most active cinnamoyl-β-amino-α-ketoamides hybrids.

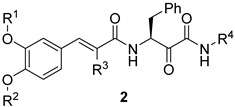					
Compound	R^1^	R^2^	R^3^	R^4^	IC_50_ (µM) ^1^
**2a**	H	H	H	benzyl	>100
**2c**	H	H	ethyl	benzyl	87.3 ± 1.2
**2d**	H	H	*n*-propyl	benzyl	45.2 ± 0.9
**2e**	H	H	*n*-propyl	4-methoxyphenethyl	37.6 ± 2.1
**2f**	H	H	*n*-butyl	benzyl	26.9 ± 1.4
**2g**	H	H	*n*-butyl	4-methoxyphenethyl	9.5 ± 0.6
**2h**	methyl	H	*n*-butyl	4-methoxyphenethyl	8.8 ± 0.4
**Erufosine**					9.8 ± 0.7

^1^ The concentration that inhibits *L. donovani* promastigotes by 50% relative to control after incubation for 3 days as calculated from the dose-response curves.

**Table 3:** In vitro evaluation of cytotoxicity on THP-1 cell of compounds **2g** and **2h**.

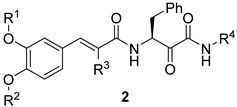					
Compound	R^1^	R^2^	R^3^	R^4^	THP-1 Cell CC_50_ (µM)
**2g**	H	H	*n*-butyl	4-methoxyphenethyl	>100
**2h**	methyl	H	*n*-butyl	4-methoxyphenethyl	>100
**Erufosine**					19.3 ± 0.8

**Table 4:** Binding scores of compounds **2g** and **2h** with the crystal structures of *L*ARG and *Ld*CALP enzymes (Kcal/mol).

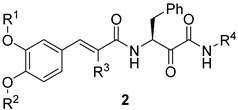						
Compound	R^1^	R^2^	R^3^	R^4^	*L*ARG	*Ld*CALP
**2g**	H	H	*n*-butyl	4-methoxyphenethyl	−7.34	−7.80
**2h**	methyl	H	*n*-butyl	4-methoxyphenethyl	−7.44	−8.10
**Rosmarinic acid**					−6.24	—
**Compound 1**					—	−8.41

## Data Availability

Data are contained within the article or Supplementary Material.

## Supplementary Materials

The following supporting information can be downloaded at: https://www.mdpi.com/article/10.3390/ph16111594/s1, Experimental procedures and protocols.
