# Supplementary material for: Design, Synthesis, and Repurposing of Rosmarinic Acid-β-Amino-α-Ketoamide Hybrids as Antileishmanial Agents
Source: Pharmaceuticals (Basel). 2023 Nov 12;16(11):1594. doi: 10.3390/ph16111594 (PMC10675174; doi:10.3390/ph16111594)
Supplement: Supplementary file 1 [file pharmaceuticals-16-01594-s001.zip › pharmaceuticals-2627401-Supplementary Materials.pdf]

Supplementary Information for

Design, synthesis, and repurposing of rosmarinic acid-β-amino-α-ketoamide hybrids as antileishmanial agents

Ahmed H.E. Hassan[1,2,*], Waleed A. Bayoumi[3], Selwan M. El-Sayed[1], Trong-Nhat Phan[5,6], Taegeun Oh[7], Gyeongpyo Ham[7], Kazem Mahmoud[8], Joo Hwan No[4], Yong Sup Lee[2,7,*]

[1] *Department of Medicinal Chemistry, Faculty of Pharmacy, Mansoura University, Mansoura 35516, Egypt.*

[2] *Medicinal Chemistry Laboratory, Department of Pharmacy, College of Pharmacy, Kyung Hee University, Seoul 02447, Republic of Korea.*

[3] *Department of Pharmaceutical Organic Chemistry, Faculty of Pharmacy, Mansoura University, Mansoura 35516, Egypt.*

[4] *Host-Parasite Research Laboratory, Institut Pasteur Korea, Seongnam-si, Gyeonggi-do 13488, Republic of Korea.*

[5] *Institute of Applied Science and Technology, School of Technology, Van Lang University, Ho Chi Minh City, Vietnam.*

[6] *Faculty of Applied Technology, School of Technology, Van Lang University, Ho Chi Minh City, Vietnam.*

[7] *Department of Fundamental Pharmaceutical Sciences, Kyung Hee University, Seoul 02447, Republic of Korea.*

[8] *Department of Pharmaceutical Chemistry, Faculty of Pharmacy, Egyptian Russian University, Badr City, Cairo11829, Egypt.*

***Corresponding authors:**

* Professor Yong Sup Lee: Medicinal Chemistry Laboratory, Department of Pharmacy, College of Pharmacy, Kyung Hee University, Seoul 02447, Republic of Korea (e-mail: kyslee@khu.ac.kr)

* Associate Professor Ahmed H.E. Hassan: Department of Medicinal Chemistry, Faculty of Pharmacy, Mansoura University, Mansoura 35516, Egypt (e-mail: ahmed_hassan@mans.edu.eg)

1. Chemistry

General

Cinnamic acid derivatives (**3a-f**) were prepared according to the published procedure [1]. The purity of biologically evaluated compounds were checked by quantitative ^{1}H NMR and showed > 95% purity.

1.1. General Procedure for amide coupling (synthesis of 5a-i)

N-(3-Dimethylaminopropyl)-*N'*-ethylcarbodiimide hydrochloride (EDC hydrochloride, 3.83 g, 19.98 mmol) was added to a cooled stirred solution (0°C) of the appropriate derivatives of cinnamic acid **3** (0.37 mmol), appropriate derivative of 3-amino-2-hydorxy-4-phenylbutanamide **4** (0.37 mmol) and 1-hydroxy benzotriazole (HOBt, 76 mg, 0.56 mmol) in DMF (5 ml). Stirring continued at an ambient temperature until reaction was complete. The reaction was quenched with water, extracted with ethyl acetate, dried over anhydrous $MgSO_4$, and evaporated under reduced pressure. Purification by column chromatography (EtOAc:Hex = 1:3 → EtOAc:Hex = 1:1) afforded the desired compounds.

(*3S*)-*N*-Benzyl-3(€-3-(3,4-bis(methoxymethoxy)phenyl)acrylamido)-2-hydroxy-4-phenylbutanamide (5a) [1]

Compound **5a** was prepared using cinnamic acid derivative **3a** and 3-amino-2-hydorxy-4-phenylbutanamide **4a** following general procedure 1.1. Yield 80%. ^{1}H NMR (400 MHz, DMSO-*d*$_6$) δ 8.44 (1H, t, *J* = 6.4 Hz, -CO-N*H*-CH$_2$-), 8.06 (1H, d, *J* = 8.7 Hz, -CO-N*H*-CH-), 7.30 (1H, s, Ar-C*H*-CH-), 7.29-7.11 (13H, m, aromatic), 6.56 (1H, d, *J* = 16 Hz, Ar-CH-C*H*-), 5.21 (4H, s, CH$_3$O-C*H*$_2$-O-), 4.50-4.43 (1H, m, -CH-C*H*(OH)-CO-), 4.39-4.26 (2H, m, -NH-C*H*$_2$-Ph), 4.13-4.11 (2H, m, -C*H*-CH$_2$-Ph, -CH-CH(O*H*)-CO-), 3.42 (3H, s, C*H*$_3$O-CH$_2$-O-), 3.40 (3H, s, C*H*$_3$O-CH$_2$-O-), 2.76-2.70 (1H, m, -CH-C*H*$_2$-Ph), 2.58 (1H, dd, *J* = 2.6, 14 Hz, -CH-C*H*$_2$-Ph).

(*3S*)-3-((*E*)-3-(3,4-Bis(methoxymethoxy)phenyl)acrylamido)-2-hydroxy-*N*-(4-methoxyphenethyl)-4-phenylbutanamide (5b) [1]

Compound **5b** was prepared using cinnamic acid derivative **3a** and 3-amino-2-hydorxy-4-phenylbutanamide **4b** following general procedure 1.1. Yield 63%. ^{1}H NMR (400 MHz, DMSO-*d*$_6$) δ 8.11 (1H, d, *J* = 8.9 Hz, -CO-N*H*-CH-), 7.94 (1H, t, *J* = 5.9 Hz, -CO-N*H*-CH$_2$-), 7.32-7.01 (9H, m, aromatic), 7.08 (1H, s, Ar-C*H*-CH-), 7.06 (1H, s, Ar-CH-C*H*-), 6.80 (1H, s, Ar-*H*5), 6.78 (1H, dd, *J* = 1.4, 8.3 Hz, Ar-*H*6), 5.20 (4H, s, CH$_3$O-C*H*$_2$-O-), 4.45-4.31 (3H, m, -CH-C*H*(O*H*)-CO-, -C*H*-CH$_2$-Ph), 3.67 (6H, s, C*H*$_3$O-CH$_2$-O-), 3.41 (3H, s, -CH$_2$-Ar-OC*H*$_3$), 3.33-3.16 (4H, m, -C*H*$_2$-C*H*$_2$-Ar-OCH$_3$), 2.88-2.82 (1H, m, -CH-C*H*$_2$-Ph), 2.74-2.62 (1H, dd, *J* = 2.6, 14 Hz, -CH-C*H*$_2$-Ph).

(*3S*)-*N*-Benzyl-3-(2-((*E*)-3,4-bis(methoxymethoxy)benzylidene)butanamido)-2-hydroxy-4-phenylbutanamide (5c) [1]

Compound **5c** was prepared using cinnamic acid derivative **3b** and 3-amino-2-hydorxy-4-phenylbutanamide **4a** following general procedure 1.1. Yield 72%. ^{1}H NMR (400 MHz, CDCl$_3$) δ 7.47 (1H, t, *J* = 6.4 Hz, -CO-N*H*-CH$_2$-), 7.34-7.24 (11H, m, -CO-N*H*-CH-, aromatic), 7.13 (1H, d, *J* = 8.4 Hz, Ar-C*H*-CH-), 7.09 (1H, s, Ar-*H*2), 6.84 (1H, dd, *J* = 1.8, 8.4 Hz, Ar-*H*6), 6.79 (1H, s, Ar-*H*5), 5.25 (2H, s, CH$_3$-C*H*$_2$-O-), 5.21 (2H, s, CH$_3$O-C*H*$_2$-O-), 4.52-4.37 (5H, m, -CH-C*H*(O*H*)-CO-, -NH-C*H*$_2$-Ph, -C*H*-CH$_2$-Ph), 3.51 (6H, s, C*H*$_3$O-CH$_2$-O-), 3.36-3.34 (1H, m, -CH-C*H*$_2$-Ph), 3.15 (1H, dd, *J* = 5.2, 13.9 Hz, -CH-C*H*$_2$-Ph), 2.43-2.36 (2H, m, -C-C*H*$_2$-CH$_3$), 0.93 (3H, t, *J* = 7.5 Hz, -C-CH$_2$-C*H*$_3$).

***N*-((2*S*)-4-(Benzylamino)-3-hydroxy-4-oxo-1-phenylbutan-2-yl)-2-((*E*)-3,4-bis(methoxymethoxy)benzylidene)pentanamide (5d) [1]**

Compound **5d** was prepared using cinnamic acid derivative **3c** and 3-amino-2-hydorxy-4-

phenylbutanamide **4a** following general procedure 1.1. Yield 94%. ^{1}H NMR (400 MHz, DMSO-*d*$_6$) δ 8.47 (1H, t, *J* = 6.2 Hz, -CO-N*H*-CH$_2$-), 7.83 (1H, d, *J* = 8.7 Hz, -CO-N*H*-CH-), 7.31-7.13 (10H, m, aromatic), 7.11 (1H, s, Ar-C*H*-C-), 7.06 (1H, s, Ar-*H*2), 6.88 (1H, dd, *J* = 1.7, 8.4 Hz, Ar-*H*6), 6.83 (1H, s, Ar-*H*5), 5.17 (2H, s, CH$_3$O-C*H*$_2$-O-), 5.15 (2H, s, CH$_3$O-C*H*$_2$-O-), 4.47-4.41 (1H, m, -CH-C*H*(OH)-CO-), 4.39-4.26 (2H, m, -NH-C*H*$_2$-Ph), 4.16-4.08 (2H, m, -C*H*-CH$_2$-Ph, -CH-CH(O*H*)-CO-), 3.40 (6H, s, C*H*$_3$O-CH$_2$-O-), 2.87-2.81 (1H, m, -CH-C*H*$_2$-Ph), 2.58 (1H, dd, *J* = 2.6, 13.8 Hz, -CH-C*H*$_2$-Ph), 2.32-2.29 (2H, t, *J* = 7.6 Hz, -C-C*H*$_2$-CH$_2$-CH$_3$), 1.24-1.19 (2H, m, -C-CH$_2$-C*H*$_2$-CH$_3$), 0.80 (3H, t, *J* = 7.2 Hz, -C-CH$_2$-CH$_2$-C*H*$_3$).

2-((*E*)-3,4-Bis(methoxymethoxy)benzylidene)-*N*-((2*S*)-3-hydroxy-4-((4-methoxyphenethyl)amino)-4-oxo-1-phenylbutan-2-yl)pentanamide (5e) [1]

Compound **5e** was prepared using cinnamic acid derivative **3c** and 3-amino-2-hydorxy-4-phenylbutanamide **4b** following general procedure 1.1. Yield 66%. ^{1}H NMR (400 MHz, DMSO-*d*$_6$) δ 7.96 (1H, t, *J* = 5.7 Hz, -CO-N*H*-CH$_2$-), 7.76 (1H, d, *J* = 8.7 Hz, -CO-N*H*-CH-), 7.30-7.06 (9H, m, aromatic), 6.89 (1H, dt, *J* = 1.6, 8.6 Hz, Ar-C*H*-C-), 6.92-6.80 (3H, m, Ar-*H*2, Ar-*H*5, Ar-*H*6), 5.19 (2H, s, CH$_3$O-C*H*$_2$-O-), 5.17 (2H, s, CH$_3$O-C*H*$_2$-O-), 4.43-4.32 (1H, m, -CH-C*H*(OH)-CO-), 4.02-4.00 (2H, m, -C*H*-CH$_2$-Ph, -CH-CH(O*H*)-CO-), 3.66 (3H, s, C*H*$_3$O-CH$_2$-O-), 3.63 (3H, s, C*H*$_3$O-CH$_2$-O-), 3.40 (3H, s, -CH$_2$-Ar-OC*H*$_3$), 3.32-3.20 (1H, m, -CH-C*H*$_2$-Ph), 2.89-2.84 (1H, m, -CH-C*H*$_2$-Ph), 2.79-2.71 (2H, m, -C*H*$_2$-CH$_2$-Ar-OCH$_3$), 2.48-2.45 (2H, m, -CH$_2$-C*H*$_2$-Ar-OCH$_3$), 1.94 (2H, s, -C*H*$_2$-CH$_2$-CH$_3$), 1.33-1.26 (2H, m, -CH$_2$-C*H*$_2$-CH$_3$), 0.83 (3H, t, *J* = 7.3 Hz, -CH$_2$-C*H*$_3$).

***N*-((2*S*)-4-(Benzylamino)-3-hydroxy-4-oxo-1-phenylbutan-2-yl)-2-((*E*)-3,4-bis(methoxymethoxy)benzylidene)hexanamide (5f) [1]**

Compound **5f** was prepared using cinnamic acid derivative **3d** and 3-amino-2-hydorxy-4-

phenylbutanamide **4a** following general procedure 1.1. Yield 81%. ^{1}H NMR (400 MHz, DMSO-*d6*) δ 8.49 (1H, t, *J* = 6.1 Hz, -CO-N*H*-CH2-), 7.84 (1H, d, *J* = 8.6 Hz, -CO-N*H*-CH-), 7.31-7.11 (10H, m, aromatic), 7.11 (1H, s, Ar-C*H*-C-), 7.06 (1H, s, Ar-*H*2), 6.88 (1H, d, *J* = 8.4 Hz, Ar-*H*6), 6.83 (1H, s, Ar-*H*5), 5.20 (2H, s, CH3-C*H2*-O-), 5.18 (2H, s, CH3O-C*H2*-O-), 4.47-4.39 (1H, m, -CH-C*H*(OH)-CO-), 4.38-4.26 (2H, m, -NH-C*H2*-Ph), 4.16-4.09 (2H, m, -C*H*-CH2-Ph, -CH-CH(O*H*)-CO-), 3.40 (6H, s, C*H3*O-CH2-O-), 2.88-2.81 (1H, m, -CH-C*H2*-Ph), 2.58 (1H, dd, *J* = 1.2, 13.6 Hz, -CH-C*H2*-Ph), 2.34-2.31 (2H, m, *J* = 7.6 Hz, -C-C*H2*-CH2-CH2-CH3), 1.21-1.18 (4H, m, -C-CH2-C*H2*-C*H2*-CH3), 0.80 (3H, t, *J* = 6.3 Hz, -C-CH2-CH2-CH2-C*H3*).

2-((*E*)-3,4-Bis(methoxymethoxy)benzylidene)-*N*-((*2S*)-3-hydroxy-4-((4-methoxyphenethyl)amino)-4-oxo-1-phenylbutan-2-yl)hexanamide (5g) [1]

Compound **5g** was prepared using cinnamic acid derivative **3d** and 3-amino-2-hydorxy-4-phenylbutanamide **4b** following general procedure 1.1. Yield 22%. ^{1}H NMR (400 MHz, DMSO-*d6*) δ 7.96 (1H, t, *J* = 5.6 Hz, -CO-N*H*-CH2-), 7.47 (1H, d, *J* = 8.8 Hz, -CO-N*H*-CH-), 7.31-7.09 (9H, m, aromatic), 6.93-6.81 (4H, m, Ar-C*H*-C-, -Ar-*H*2, Ar-*H*6, Ar-*H*5), 5.20 (2H, s, CH3O-C*H2*-O-), 5.15 (2H, s, CH3O-C*H2*-O-), 4.41-4.33 (1H, m, -CH-C*H*(OH)-CO-), 4.06-4.02 (2H, m, -C*H*-CH2-Ph, -CH-CH(O*H*)-CO-), 3.64 (3H, s, -CH2-Ar-OC*H3*), 3.40 (6H, s, C*H3*O-CH2-O-), 3.28-3.21 (1H, m, -CH-C*H2*-Ph), 2.89-2.83 (1H, m, -CH-C*H2*-Ph), 2.79-2.76 (4H, m, -C*H2*-C*H2*-Ar-OCH3), 2.45-2.41 (2H, m, -C*H2*-CH2-CH2-CH3), 1.23-1.20 (4H, m, -CH2-C*H2*-C*H2*-CH3), 0.79 (3H, t, *J* = 6.5 Hz, -CH2-CH2-CH2-C*H3*).

***N*-((*2S*)-3-Hydroxy-4-((4-methoxyphenethyl)amino)-4-oxo-1-phenylbutan-2-yl)-2-((*E*)-4-methoxy-3-(methoxymethoxy)benzylidene)hexanamide (5h) [1]**

Compound **5h** was prepared using cinnamic acid derivative **3e** and 3-amino-2-hydorxy-4-phenylbutanamide **4b** following general procedure 1.1. Yield 71%. ^{1}H NMR (400 MHz, DMSO-

d_6) δ 7.99 (1H, t, J = 5.6 Hz, -CO-N$\underline{H}$-CH$_2$-), 7.75 (1H, d, J = 7.4 Hz, -CO-N$\underline{H}$-CH-), 7.30-7.08 (9H, m, aromatic), 6.89-6.80 (4H, s, Ar-C$\underline{H}$-C-, aromatic), 5.17 (2H, s, CH$_3$O-C$\underline{H_2}$-O-), 4.42-4.35 (1H, m, -CH-C$\underline{H}$(OH)-CO-), 4.01-3.90 (2H, m, -C$\underline{H}$-CH$_2$-Ph, -CH-CH(O$\underline{H}$)-CO-), 3.76 (3H, s, C$\underline{H_3}$O-CH$_2$-O-), 3.65 (3H, s, -CH$_2$-Ar-OC$\underline{H_3}$), 3.34-3.21 (2H, m, -C$\underline{H_2}$-CH$_2$-Ar-OCH$_3$), 2.88-2.85 (1H, m, -CH-C$\underline{H_2}$-Ph), 2.77-2.62 (3H, m, -CH$_2$-C$\underline{H_2}$-Ar-OCH$_3$, -CH-C$\underline{H_2}$-Ph), 2.36-2.35 (2H, m, -C$\underline{H_2}$-CH$_2$-CH$_2$-CH$_3$), 1.25-1.19 (4H, s, -CH$_2$-C$\underline{H_2}$-C$\underline{H_2}$-CH$_2$-), 0.81 (3H, t, J = 6.5 Hz, -CH$_2$-CH$_2$-CH$_2$-C$\underline{H_3}$).

2-((*E*)-3,4-Dimethoxybenzylidene)-*N*-((*2S*)-3-hydroxy-4-((4-methoxyphenethyl)amino)-4-oxo-1-phenylbutan-2-yl)hexanamide (5i) [1]

Compound **5a** was prepared using cinnamic acid derivative **3f** and 3-amino-2-hydorxy-4-phenylbutanamide **4b** following general procedure 1.1. Yield 64%. ^{1}H NMR (400 MHz, DMSO-d_6) δ 8.00 (1H, t, J = 5.7 Hz, -CO-N$\underline{H}$-CH$_2$-), 7.73 (1H, d, J = 8.8 Hz, -CO-N$\underline{H}$-CH-), 7.30-7.08 (9H, m, aromatic), 6.99 (1H, d, J = 7.9 Hz, Ar-C$\underline{H}$-C-), 6.88-6.80 (3H, m, aromatic), 4.44-4.35 (1H, m, -CH-C$\underline{H}$(OH)-CO-), 4.02-4.00 (1H, m, -C$\underline{H}$-CH$_2$-Ph), 3.90-3.89 (1H, m, -CH-CH(O$\underline{H}$)-CO-), 3.77 (3H, s, -CH-Ar-OC$\underline{H_3}$), 3.75 (3H, s, -CH-Ar-OC$\underline{H_3}$), 3.68 (3H, s, -CH$_2$-Ar-OC$\underline{H_3}$), 3.32-3.19 (2H, m, -C$\underline{H_2}$-CH$_2$-Ar-OCH$_3$), 2.89-2.84 (1H, dd, J = 6.4, 13.6 Hz, -C-C$\underline{H_2}$-Ph), 2.77-2.62 (3H, m, -CH$_2$-C$\underline{H_2}$-Ar-OCH$_3$, -CH-C$\underline{H_2}$-Ph), 2.37-2.33 (2H, m, -C$\underline{H_2}$-CH$_2$-CH$_2$-CH$_3$), 1.25-1.20 (4H, m, -CH$_2$-C$\underline{H_2}$-C$\underline{H_2}$-CH$_3$), 0.82-0.78 (3H, m, -CH$_2$-CH$_2$-CH$_2$-C$\underline{H_3}$).

1.2. General Procedure for Dess-Martin periodinane oxidation (synthesis o' 2'a-h and 2i)

Dess–Martin periodinane (262 mg, 0.62 mmol) was added to a cooled solution (0°C) of the appropriate derivative of compound **5** (0.28 mmol) in DMF (3 ml). Stirring continued at an ambient temperature until reaction was complete. The reaction was quenched by 10% $Na_2S_2O_3$. The formed precipitate was collected by filtration to afford the desired compounds.

_(S,E)_-_N_-Benzyl-3-(3-(3,4-bis(methoxymethoxy)phenyl)acrylamido)-2-oxo-4-phenylbutanamide (2'a) [1]

Compound **2'a** was obtained from compound **5a** following general procedure 1.2. Yield 97%. ^{1}H NMR (400 MHz, $CDCl_3$) δ 7.31 (1H, s, Ar-C*H*-CH-), 7.30-7.09 (13H, m, aromatic), 6.57 (1H, d, *J* = 15 Hz, Ar-CH-C*H*-), 5.60-5.57 (1H, m, -C*H*-CH_2-Ph), 5.21 (4H, s, CH_3O-CH_2-O-), , 4.38-4.27 (2H, m, -NH-CH_2-Ph), 3.45 (1H, dd, *J* = 5.4, 14.1 Hz, -CH-CH_2-Ph), 3.43 (3H, s, CH_3O-CH_2-O-), 3.40 (3H, s, CH_3O-CH_2-O-), 3.25-3.20 (2H, m, -CH-CH_2-Ph).

_(S,E)_-3-(3-(3,4-Bis(methoxymethoxy)phenyl)acrylamido)-_N_-(4-methoxyphenethyl)-2-oxo-4-phenylbutanamide (2'b) [1]

Compound **2'b** was obtained from compound **5b** following general procedure 1.2. Yield 94%. ^{1}H NMR (400 MHz, DMSO-*d6*) δ 8.83 (1H, t, *J* = 5.6 Hz, -CO-N*H*-CH_2-), 8.52 (1H, d, *J* = 7.3 Hz, -CO-N*H*-CH-), 7.33-7.11 (9H, m, aromatic), 6.85 (1H, s, Ar-C*H*-CH-), 6.84 (1H, s, Ar-*H*2), 6.83 (1H, s, Ar-CH-C*H*-), 6.73 (1H, d, *J* = 8.4 Hz, Ar-*H*5), 6.39 (1H, dd, *J* = 2, 8.4 Hz, Ar-*H*6), 5.37-5.32 (1H, m, -C*H*-CH_2-Ph), 5.25 (2H, s, CH_3O-CH_2-O-), 5.23 (2H, s, CH_3O-CH_2-O-), 3.67 (3H, s, -CH_2-Ar-OCH_3), 3.41 (6H, s, CH_3O-CH_2-O-), 3.34-3.27 (1H, m, -CH-CH_2-Ph), 3.08 (1H, dd, *J* = 3.6, 14.1 Hz, -CH-CH_2-Ph), 2.77-2.66 (4H, m, -CH_2-CH_2-Ar-OCH_3).

_(S,E)_-_N_-Benzyl-3-(2-(3,4-bis(methoxymethoxy)benzylidene)butanamido)-2-oxo-4-phenylbutanamide (2'c) [1]

Compound **2'c** was obtained from compound **5c** following general procedure 1.2. Yield 47%. ^{1}H NMR (400 MHz, $CDCl_3$) δ 7.38-7.24 (10H, m, aromatic), 7.15 (1H, s, Ar-C*H*-C-), 7.13 (1H, s, Ar-*H*2), 7.02 (1H, s, Ar-*H*5), 6.90 (1H, dd, *J* = 1.9, 8.4 Hz, Ar-*H*6), 5.60-5.56 (1H, m, -C*H*-CH_2-Ph), 5.24 (2H, s, CH_3O-CH_2-O-), 5.21 (2H, s, CH_3O-CH_2-O-), 4.53-4.50 (2H, m, -NH-CH_2-Ph), 3.51 (6H, s, CH_3O-CH_2-O-), 3.44 (1H, dd, *J* = 5.4, 14.1 Hz, -CH-CH_2-Ph), 3.25-3.19 (1H, m, -

CH-C*H*$_2$-Ph), 2.51-2.46 (2H, m, -C*H*$_2$-CH$_3$), 1.05 (3H, t, *J* = 7.5 Hz, -CH$_2$-C*H*$_3$).

***(S,E)*-*N*-(4-(Benzylamino)-3,4-dioxo-1-phenylbutan-2-yl)-2-(3,4-bis(methoxymethoxy)benzylidene)pentanamide (2'd) [1]**

Compound **2'd** was obtained from compound **5d** following general procedure 1.2. Yield 89%. ^{1}H NMR (400 MHz, DMSO-*d*$_6$) δ 9.23 (1H, t, *J* = 6.0 Hz, -CO-N*H*-CH$_2$-), 8.48 (1H, d, *J* = 7.2 Hz, -CO-N*H*-CH-), 7.32-7.25 (10H, m, aromatic), 7.14 (1H, s, Ar-C*H*-C-), 7.09 (1H, s, Ar-*H*2), 6.93 (2H, s, Ar-*H*5,6), 5.21 (2H, s, CH$_3$O-C*H*$_2$-O-), 5.18 (2H, s, CH$_3$O-C*H*$_2$-O-), 5.20-5.14 (1H, m, -C*H*-CH$_2$-Ph), 4.35-4.33 (2H, m, -NH-C*H*$_2$-Ph), 3.41 (6H, s, C*H*$_3$O-CH$_2$-O-), 3.19 (1H, dd, *J* = 4.0, 13.6 Hz, -CH-C*H*$_2$-Ph), 2.97-2.86 (1H, m, -CH-C*H*$_2$-Ph), 2.36 (2H, t, *J* = 7.5 Hz, -C*H*$_2$-CH$_2$-CH$_3$), 1.33-1.28 (2H, m, -CH$_2$-C*H*$_2$-CH$_3$), 0.84 (3H, t, *J* = 7.2 Hz, -CH$_2$-CH$_2$-C*H*$_3$).

***(S,E)*-2-(3,4-Bis(methoxymethoxy)benzylidene)-*N*-(4-(4-methoxyphenethylamino)-3,4-dioxo-1-phenylbutan-2-yl)pentanamide (2'e) [1]**

Compound **2'e** was obtained from compound **5e** following general procedure 1.2. Yield 97%. ^{1}H NMR (400 MHz, DMSO-*d*$_6$) δ 8.80 (1H, t, *J* = 5.7 Hz, -CO-N*H*-CH$_2$-), 8.43 (1H, d, *J* = 7.3 Hz, -CO-N*H*-CH-), 7.33-7.09 (9H, m, aromatic), 6.93-6.83 (4H, m, aromatic), 5.21 (2H, s, CH$_3$O-C*H*$_2$-O-), 5.18 (2H, s, CH$_3$O-C*H*$_2$-O-), 5.20-5.16 (1H, m, -C*H*-CH$_2$-Ph), 3.68 (3H, s, -CH$_2$-Ar-OC*H*$_3$), 3.40 (6H, s, C*H*$_3$O-CH$_2$-O-), 3.08 (1H, dd, *J* = 3.7, 13.7 Hz, -CH-C*H*$_2$-Ph), 2.85-2.79 (1H, m, -CH-C*H*$_2$-Ph), 2.73-2.67 (2H, m, -C*H*$_2$-CH$_2$-Ar-OCH$_3$), 2.37-2.33 (2H, m, -CH$_2$-C*H*$_2$-Ar-OCH$_3$), 2.09-2.07 (2H, m, -C*H*$_2$-CH$_2$-CH$_3$) 1.33-1.28 (2H, m, -CH$_2$-C*H*$_2$-CH$_3$), 0.84 (3H, t, *J* = 7.2 Hz, -CH$_2$-CH$_2$-C*H*$_3$).

***(S,E)*-*N*-(4-(Benzylamino)-3,4-dioxo-1-phenylbutan-2-yl)-2-(3,4-bis(methoxymethoxy)benzylidene)hexanamide (2'f) [1]**

Compound **2'f** was obtained from compound **5f** following general procedure 1.2. Yield 99%. ^{1}H NMR (400 MHz, DMSO-*d6*) δ 9.22 (1H, t, *J* = 6.2 Hz, -CO-N*H*-CH2-), 8.45 (1H, d, *J* = 7.2 Hz, -CO-N*H*-CH-), 7.34-7.18 (10H, m, aromatic), 7.15 (1H, s, Ar-C*H*-C-), 7.12 (1H, s, Ar-*H*2), 7.09 (1H, d, *J* = 8 Hz, Ar-*H*5), 6.91 (1H, d, *J* = 10 Hz, Ar-*H*6), 5.24-5.20 (1H, m, -C*H*-CH2-Ph), 5.23 (2H, s, CH3O-C*H2*-O-), 5.18 (2H, s, CH3O-C*H2*-O-), 4.40-4.30 (2H, m, -NH-C*H2*-Ph), 3.41 (6H, s, C*H3*O-CH2-O-), 3.19 (1H, dd, *J* = 4.1, 13.7 Hz, -CH-C*H2*-Ph), 2.95-2.87 (1H, m, -CH-C*H2*-Ph), 2.42-2.37 (2H, m, -C*H2*-CH2-CH2-CH3), 1.26-1.24 (4H, m, -CH2-C*H2*-C*H2*-CH3), 0.81 (3H, t, *J* = 6.7 Hz, -CH2-CH2-CH2-C*H3*).

***(S,E)*-2-(3,4-Bis(methoxymethoxy)benzylidene)-*N*-(4-(4-methoxyphenethylamino)-3,4-dioxo-1-phenylbutan-2-yl)hexanamide (2'g) [1]**

Compound **2'g** was obtained from compound **5g** following general procedure 1.2. Yield 23%. ^{1}H NMR (400 MHz, DMSO-*d6*) δ 8.81 (1H, t, *J* = 5.7 Hz, -CO-N*H*-CH2-), 8.43 (1H, d, *J* = 7.4 Hz, -CO-N*H*-CH-), 7.33-7.09 (9H, m, aromatic), 6.93 (1H, d, J = 8.5 Hz, Ar-*H*6), 6.88 (1H, s, Ar-C*H*-C-), 6.85 (1H, s, -Ar-*H*2), 6.83 (1H, s, Ar-*H*5), 5.21 (2H, s, CH3O-C*H2*-O-), 5.18 (2H, s, CH3O-C*H2*-O-), 5.20-5.16 (1H, m, -C*H*-CH2-Ph), 3.68 (3H, s, -CH2-Ar-OC*H3*), 3.40 (6H, s, C*H3*O-CH2-O-), 3.08 (1H, dd, *J* = 2.0, 12.4 Hz, -CH-C*H2*-Ph), 2.85-2.79 (1H, m, -CH-C*H2*-Ph), 2.74-2.68 (2H, m, -C*H2*-CH2-Ar-OCH3), 2.41-2.37 (2H, m, -CH2-C*H2*-Ar-OCH3) 2.32-2.30 (2H, m, -C*H2*-CH2-CH2-CH3), 1.25-1.24 (4H, m, -CH2-C*H2*-C*H2*-CH2-), 0.83 (3H, t, *J* = 6.4 Hz, -CH2-CH2-CH2-C*H3*).

***(S,E)*-2-(4-Methoxy-3-(methoxymethoxy)benzylidene)-*N*-(4-(4-methoxyphenethylamino)-3,4-dioxo-1-phenylbutan-2-yl)hexanamide (2'h) [1]**

Compound **2'h** was obtained from compound **5h** following general procedure 1.2. Yield 96%. ^{1}H NMR (400 MHz, DMSO-*d6*) δ 8.82 (1H, t, *J* = 5.8 Hz, -CO-N*H*-CH2-), 8.42 (1H, d, *J* = 7.4 Hz, -CO-N*H*-CH-), 7.31-7.22 (9H, m, aromatic), 6.95 (1H, s, Ar-C*H*-C-), 6.89 (1H, s, Ar-*H*2), 6.81

(2H, d, J = 11.6 Hz, Ar-*H*5,6), 5.25-5.21 (1H, m, -C*H*-CH_2-Ph), 5.18 (2H, s, CH_3O-C*H_2*-O-), 3.77 (3H, s, -CH-Ar-OC*H_3*), 3.69 (3H, s, -CH_2-Ar-OC*H_3*), 3.08 (1H, dd, J = 3.4, 13.7 Hz, -CH-C*H_2*-Ph), 2.86-2.81 (1H, m, -CH-C*H_2*-Ph), 2.74-2.70 (2H, m, -C*H_2*-CH_2-Ar-OCH_3), 2.41-2.34 (2H, m, -CH_2-C*H_2*-Ar-OCH_3) 2.32-2.30 (2H, m, -C*H_2*-CH_2-CH_2-CH_3), 1.26 (2H, s, -C-CH_2-C*H_2*-CH_2-CH_2-), 1.25 (2H, s, -CH_2-CH_2-C*H_2*-CH_3), 0.81 (3H, t, J = 6.8 Hz, -CH_2-CH_2-CH_2-C*H_3*).

***(S,E)*-2-(3,4-Dimethoxybenzylidene)-*N*-(4-(4-methoxyphenethylamino)-3,4-dioxo-1-phenylbutan-2-yl)hexanamide (2i) [1]**

Compound **2i** was obtained from compound **5i** following general procedure 1.2. Yield 96%. ^{1}H NMR (400 MHz, DMSO-*d_6*) δ 8.80 (1H, t, J = 5.6 Hz, -CO-N*H*-CH_2-), 8.39 (1H, d, J = 7.2 Hz, -CO-N*H*-CH-), 7.31-7.12 (9H, m, aromatic), 6.93 (1H, s, Ar-C*H*-C-), 6.89-6.83 (3H, m, aromatic), 5.24-5.21 (1H, m, -NH-C*H*-CH_2-), 3.77 (3H, s, -CH-Ar-OC*H_3*), 3.75 (3H, s, -CH-Ar-OC*H_3*), 3.68 (3H, s, -CH_2-Ar-OC*H_3*), 3.09 (1H, dd, J = 3.6, 14 Hz, -C-C*H_2*-Ph), 2.86-2.80 (1H, m, -C-C*H_2*-Ph), 2.74-2.70 (2H, m, -NH-C*H_2*-CH_2-), 2.41 (2H, brs, -CH_2-C*H_2*-Ar-), 2.32-2.30 (2H, m, -C-C*H_2*-CH_2-), 1.27 (2H, s, -CH_2-C*H_2*-CH_2-CH_3), 1.26 (2H, s, -CH_2-CH_2-C*H_2*-CH_3), 0.82 (3H, t, J = 6.4 Hz, -CH_2-CH_2-C*H_3*); ^{13}C NMR (DMSO) δ 196.8, 169.5, 160.7, 157.7, 148.5, 148.3, 138.0, 135.7, 132.2, 130.8, 129.6, 129.6, 129.0, 129.0, 128.2, 128.2, 126.4, 121.6, 113.7, 113.7, 112.1, 111.5, 56.4, 56.1, 56.1, 55.8, 40.9, 35.6, 35.4, 28.9, 23.8, 22.4, 14.2.

1.3. General Procedure for MOM-deprotection (2a–h):

The appropriate derivative of compound **2'** (0.26 mmol) was refluxed in methanolic HCl (10 mL, 1%) solution until complete deprotection. After cooling to an ambient temperature, the mixture was evaporated under reduced pressure, extracted with ethyl acetate/water, dried over anhydrous $MgSO_4$, and the solvent was removed under reduced pressure to afford the desired compounds.

(*S,E*)-*N*-Benzyl-3-(3-(3,4-dihydroxyphenyl)acrylamido)-2-oxo-4-phenylbutanamide (2a) [1]

Compound **2a** was obtained from compound **2'a** following general procedure 1.3. Yield 71%. ^{1}H NMR (400 MHz, DMSO-d_6) δ 9.23 (1H, t, *J* = 6.2 Hz, -CO-N*H*-CH$_2$-), 9.14 (1H, brs, O*H*), 9.05 (1H, brs, O*H*), 8.49 (1H, d, *J* = 7.0 Hz, -CO-N*H*-CH-), 7.30-7.18 (10H, m, aromatic), 6.94 (1H, s, Ar-C*H*-CH-), 6.84 (1H, s, Ar-*H*2), 6.82 (1H, s, Ar-CH-C*H*-), 6.74 (1H, d, *J* = 8.2 Hz, Ar-*H*5), 6.38 (1H, dd, *J* = 1.8, 8.2 Hz, Ar-*H*6), 5.24-5.23 (1H, m, -C*H*-CH$_2$-Ph), 4.34 (2H, m, -NH-C*H$_2$*-Ph), 3.17 (1H, dd, *J* = 4.2, 13.8 Hz, -CH-C*H$_2$*-Ph), 2.88-2.82 (1H, m, -CH-C*H$_2$*-Ph); ^{13}C NMR (DMSO) δ 196.6, 165.6, 161.3, 147.5, 145.5, 140.5, 140.1, 138.4, 130.0, 129.0, 129.0, 128.2, 128.2, 128.1, 128.1, 127.2, 127.2, 126.8, 126.1, 120.6, 117.2, 115.7, 113.8, 55.8, 42.0, 35.3.

(*S,E*)-3-(3-(3,4-Dihydroxyphenyl)acrylamido)-*N*-(4-methoxyphenethyl)-2-oxo-4-phenylbutanamide (2b) [1]

Compound **2b** was obtained from compound **2'b** following general procedure 1.3. Yield 83%. ^{1}H NMR (400 MHz, DMSO-d_6) δ 9.42 (1H, brs, O*H*), 9.18 (1H, brs, O*H*), 8.85 (1H, t, *J* = 5.6 Hz, -CO-N*H*-CH$_2$-), 8.43 (1H, d, *J* = 7.2 Hz, -CO-N*H*-CH-), 7.31-7.11 (9H, m, aromatic), 6.93 (1H, s, Ar-C*H*-CH-), 6.84 (1H, s, Ar-*H*2), 6.83 (1H, s, Ar-CH-C*H*-), 6.73 (1H, d, *J* = 8.4 Hz, Ar-*H*5), 6.39 (1H, dd, *J* = 2, 8.4 Hz, Ar-*H*6), 5.33-5.28 (1H, m, -C*H*-CH$_2$-Ph), 3.68 (3H, s, -CH$_2$-Ar-OC*H$_3$*), 3.33-3.28 (1H, m, -CH-C*H$_2$*-Ph), 3.08 (1H, dd, *J* = 3.6, 14 Hz, -CH-C*H$_2$*-Ph), 2.76-2.71 (4H, m, -C*H$_2$*-C*H$_2$*-Ar-OCH$_3$); ^{13}C NMR (DMSO) δ 197.1, 166.0, 161.0, 158.1, 148.0, 146.0, 140.6, 138.0, 131.3, 129.4, 129.4, 129.4, 128.8, 128.8, 127.0, 127.0, 126.5, 121.1, 117.7, 116.2, 114.2, 114.1, 114.1, 56.0, 55.4, 40.9, 35.6, 34.1.

(*S,E*)-*N*-Benzyl-3-(2-(3,4-dihydroxybenzylidene)butanamido)-2-oxo-4-phenylbutanamide (2c) [1]

Compound **2c** was obtained from compound **12'c** following general procedure 1.3. Yield 82%. ^{1}H NMR (400 MHz, DMSO-d_6) δ 9.17 (1H, t, *J* = 6.2 Hz, -CO-N*H*-CH$_2$-), 9.14 (1H, brs, O*H*), 9.05

(1H, brs, O*H*), 8.41 (1H, d, *J* = 7.0 Hz, -CO-N*H*-CH-), 7.31-7.16 (10H, m, aromatic), 6.85 (1H, s, Ar-C*H*-C-), 6.78 (1H, s, Ar-*H*2), 6.76 (1H, d, *J* = 8.2 Hz, Ar-*H*5), 6.63 (1H, dd, *J* = 1.8, 8.2 Hz, Ar-*H*6), 5.20-5.15 (1H, m, -C*H*-CH_2-Ph), 4.39-4.29 (2H, m, -NH-CH_2-Ph), 3.17 (1H, dd, *J* = 4.2, 13.8 Hz, -CH-CH_2-Ph), 2.95-2.89 (1H, m, -CH-CH_2-Ph), 2.44-2.38 (2H, m, -CH_2-CH_3), 0.82 (3H, t, *J* = 7.3 Hz, -CH_2-CH_3); ^{13}C NMR (DMSO) δ 196.8, 169.4, 161.2, 145.6, 145.0, 138.5, 138.0, 135.1, 132.6, 129.0, 128.2, 128.2, 128.1, 128.1, 127.2, 127.2, 126.9, 126.9, 126.9, 126.8, 126.4, 120.9, 116.1, 115.6, 56.2, 42.0, 34.6, 20.4, 14.1.

(*S,E*)-*N*-(4-(Benzylamino)-3,4-dioxo-1-phenylbutan-2-yl)-2-(3,4-dihydroxybenzylidene)pentanamide (2d) [1]

Compound **2d** was obtained from compound **2'd** following general procedure 1.3. Yield 83%. ^{1}H NMR (400 MHz, DMSO-*d*6) δ 9.22 (1H, t, *J* = 6.3 Hz, -CO-N*H*-CH_2-), 9.17 (1H, brs, O*H*), 9.09 (1H, brs, O*H*), 8.39 (1H, d, *J* = 7.1 Hz, -CO-N*H*-CH-), 7.32-7.20 (10H, m, aromatic), 6.84 (1H, s, Ar-C*H*-C-), 6.77 (1H, s, Ar-*H*2), 6.76 (1H, d, *J* = 8.2 Hz, Ar-*H*5), 6.62 (1H, dd, *J* = 1.6, 8.4 Hz, Ar-*H*6), 5.20-5.14 (1H, m, -C*H*-CH_2-Ph), 4.39-4.29 (2H, m, -NH-CH_2-Ph), 3.17 (1H, dd, *J* = 4.0, 13.7 Hz, -CH-CH_2-Ph), 2.94-2.88 (1H, m, -CH-CH_2-Ph), 2.36 (2H, t, *J* = 7.5 Hz, -CH_2-CH_2-CH_3), 1.35-1.26 (2H, m, -CH_2-CH_2-CH_3), 0.82 (3H, t, *J* = 7.2 Hz, -CH_2-CH_2-CH_3); ^{13}C NMR (DMSO) δ 196.8, 169.8, 161.1, 145.6, 145.0, 138.5, 134.1, 132.9, 129.0, 128.2, 128.2, 128.1, 128.1, 127.3, 127.3, 126.9, 126.9, 126.4, 121.0, 121.0, 116.1, 115.6, 56.2, 42.0, 34.6, 29.1, 21.4, 13.9.

(*S,E*)-2-(3,4-Dihydroxybenzylidene)-*N*-(4-(4-methoxyphenethylamino)-3,4-dioxo-1-phenylbutan-2-yl)pentanamide (2e) [1]

Compound **2e** was obtained from compound **2'e** following general procedure 1.3. Yield 69%. ^{1}H NMR (400 MHz, DMSO-*d*6) δ 9.20 (1H, brs, O*H*), 9.12 (1H, brs, O*H*), 8.78 (1H, t, *J* = 6 Hz, -CO-N*H*-CH_2-), 8.35 (1H, d, *J* = 7.2 Hz, -CO-N*H*-CH-), 7.33-7.11 (9H, m, aromatic), 6.86 (1H, s, Ar-

C*H*-C-), 6.84 (1H, s, Ar-*H*2), 6.76 (1H, d, *J* = 8.4 Hz, Ar-*H*5), 6.67 (1H, dd, *J* = 2, 8.4 Hz, Ar-*H*6), 5.23-5.18 (1H, m, -C*H*-CH$_2$-Ph), 3.68 (3H, s, -CH$_2$-Ar-OC*H*$_3$), 3.08 (1H, dd, *J* = 3.6, 14 Hz, -CH-C*H*$_2$-Ph), 2.85-2.79 (1H, m, -CH-C*H*$_2$-Ph), 2.73-2.69 (2H, m, -NH-C*H*$_2$-CH$_2$-), 2.43-2.40 (2H, m, -CH$_2$-C*H*$_2$-Ar-OCH$_3$), 2.38-2.34 (2H, m, -C*H*$_2$-CH$_2$-CH$_3$), 1.33-1.28 (1H, m, -CH$_2$-C*H*$_2$-CH$_3$), 0.85 (3H, t, *J* = 7.2 Hz, -CH$_2$-CH$_2$-C*H*$_3$); ^{13}C NMR (DMSO) δ 196.8, 169.8, 160.9, 157.7, 145.5, 145.1, 138.1, 136.7, 134.3, 132.7, 130.9, 129.5, 129.5, 129.0, 129.0, 128.2, 128.2, 126.4, 120.9, 116.2, 115.6, 113.7, 113.7, 56.1, 54.9, 40.3, 34.5, 33.7, 29.2, 21.4, 13.9.

(*S,E*)-*N*-(4-(Benzylamino)-3,4-dioxo-1-phenylbutan-2-yl)-2-(3,4-dihydroxybenzylidene)hexanamide (2f) [1]

Compound **2f** was obtained from compound **2'f** following general procedure 1.3. Yield 69%. ^{1}H NMR (400 MHz, DMSO-*d*$_6$) δ 9.27 (1H, t, *J* = 6.4 Hz, -CO-N*H*-CH$_2$-), 9.22 (1H, brs, O*H*), 9.11 (1H, brs, O*H*), 8.42 (1H, d, *J* = 7.2 Hz, -CO-N*H*-CH-), 7.32-7.20 (10H, m, aromatic), 6.83 (1H, s, Ar-C*H*-C-), 6.77 (1H, s, Ar-*H*2), 6.75 (1H, d, *J* = 8 Hz, Ar-*H*5), 6.62 (1H, dd, *J* = 1.6, 8.4 Hz, Ar-*H*6), 5.20-5.15 (1H, m, -C*H*-CH2-Ph), 4.35-4.31 (2H, m, -NH-C*H*$_2$-Ph), 3.17 (1H, dd, *J* = 4.0, 14.0 Hz, -CH-C*H*$_2$-Ph), 2.93-2.87 (1H, m, -CH-C*H*$_2$-Ph), 2.38-2.36 (2H, m, -C*H*$_2$-CH$_2$-CH$_2$-CH$_3$), 1.25 (2H, s, -CH$_2$-C*H*$_2$-CH$_2$-CH$_3$), 1.17 (2H, t, *J* = 7.2 Hz, -CH$_2$-CH$_2$-C*H*$_2$-CH$_3$), 0.82 (3H, t, *J* = 7.2 Hz, -CH$_2$-CH$_2$-CH$_2$-C*H*$_3$); ^{13}C NMR (DMSO) δ 197.3, 170.2, 161.6, 146.0, 145.5, 139.0, 138.5, 134.7, 133.2, 129.5, 128.8, 128.8, 128.7, 128.7, 127.7, 127.7, 127.4, 127.4, 126.9, 121.4, 116.6, 116.1, 56.7, 42.5, 35.0, 30.8, 27.4, 22.7, 14.3.

(*S,E*)-2-(3,4-Dihydroxybenzylidene)-*N*-(4-(4-methoxyphenethylamino)-3,4-dioxo-1-phenylbutan-2-yl)hexanamide (2g) [1]

Compound **2g** was obtained from compound **2'g** following general procedure 1.3. Yield 62%. ^{1}H NMR (400 MHz, DMSO-*d*$_6$) δ 9.22 (1H, brs, O*H*), 9.11 (1H, brs, O*H*), 8.80 (1H, t, *J* = 5.6 Hz, -

CO-N*H*-CH_2-), 8.34 (1H, d, J = 7.3 Hz, -CO-N*H*-CH-), 7.32-7.12 (9H, m, aromatic), 6.89 (1H, s, Ar-C*H*-C-), 6.84 (1H, s, Ar-*H*2), 6.75 (1H, d, J = 8.8 Hz, Ar-*H*5), 6.61 (1H, d, J = 8.2 Hz, Ar-*H*6), 5.21-5.16 (1H, m, -C*H*-CH_2-Ph), 3.68 (3H, s, -CH_2-Ar-OCH_3), 3.07 (1H, dd, J = 3.3, 13.8 Hz, -CH-CH_2-Ph), 2.85-2.75 (1H, m, -CH-CH_2-Ph), 2.74-2.66 (2H, m, -CH_2-CH_2-Ar-OCH_3), 2.42-2.37 (2H, m, -CH_2-CH_2-Ar-OCH_3) 2.32-2.30 (2H, m, -CH_2-CH_2-CH_2-CH_3), 1.27 (2H, s, -CH_2-CH_2-CH_2-CH_3), 1.26 (2H, s, -CH_2-CH_2-CH_2-CH_3), 0.82 (3H, t, J = 6.8 Hz, -CH_2-CH_2-CH_2-CH_3); ^{13}C NMR (DMSO) δ 197.3, 167.5, 161.3, 158.2, 146.0, 145.5, 138.6, 134.9, 131.3, 130.1, 129.5, 129.1, 129.1, 128.7, 128.7, 127.4, 126.9, 121.4, 116.6, 116.1, 114.2, 114.2, 56.7, 55.4, 40.8, 34.8, 34.2, 27.4, 23.7, 22.9, 14.3.

(*S,E*)-2-(3-Hydroxy-4-methoxybenzylidene)-*N*-(4-(4-methoxyphenethylamino)-3,4-dioxo-1-phenylbutan-2-yl)hexanamide (2h) [1]

Compound **2h** was obtained from compound **2'h** following general procedure 1.3. Yield 87%. ^{1}H NMR (400 MHz, DMSO-d_6) δ 9.35 (1H, brs, O*H*), 8.79 (1H, t, J = 6 Hz, -CO-N*H*-CH_2-), 8.35 (1H, d, J = 7.2 Hz, -CO-N*H*-CH-), 7.31-7.12 (9H, m, aromatic), 6.91 (1H, s, Ar-C*H*-C-), 6.85 (1H, s, Ar-*H*2), 6.81 (1H, d, J = 8 Hz, Ar-*H*5), 6.69 (1H, dd, J = 2.0, 8.4 Hz, Ar-*H*6), 5.22-5.18 (1H, m, -C*H*-CH2-Ph), 3.76 (3H, s, -CH-Ar-OCH_3), 3.68 (3H, s, -CH_2-Ar-OCH_3), 3.08 (1H, dd, J = 3.6, 13.6 Hz, -CH-CH_2-Ph), 2.86-2.80 (1H, m, -CH-CH_2-Ph), 2.73-2.67 (2H, m, -CH_2-CH_2-Ar-OCH_3), 2.43-2.40 (2H, m, -CH_2-CH_2-Ar-OCH_3) 2.32-2.30 (2H, m, -CH_2-CH_2-CH_2-CH_3), 1.27 (2H, s, -CH_2-CH_2-CH_2-CH_3), 1.26 (2H, s, -CH_2-CH_2-CH_2-CH_3), 0.82 (3H, t, J = 6.8 Hz, -CH_2-CH_2-CH_2-CH_3); ^{13}C NMR (DMSO) δ 196.8, 169.6, 160.9, 157.7, 147.3, 146.6, 138.0, 134.8, 132.5, 130.8, 129.6, 129.6, 129.0, 129.0, 128.2, 128.2, 126.9, 126.4, 122.2, 115.5, 113.7, 113.7, 112.8, 56.1, 55.4, 54.9, 40.3, 34.5, 33.7, 27.0, 22.2, 22.2, 13.8.

2. Biological evaluations

2.1. In vitro *L. donovani* promastigotes-based evaluation model [2-4]

2.1.1. Cell culture of parasite

L. donovani MHOM/SD/62/1S-CL2D parasites (Department of PIV, Institut Pasteur, Paris, France) were cultured as promastigotes at 28 °C in M199 medium (Sigma-Aldrich, St. Louis, MO, USA) with 40 mM HEPES, 0.1 mM adenine, 0.0001% biotin, and 4.62 mM NaHCO3 supplemented with 10% foetal bovine serum (FBS, Gibco, Carlsbad, CA, USA), 100 m/mL penicillin (Gibco), and 100 mg/mL streptomycin (Gibco). Parasites were sub-cultured every 3 or 4 days and maintained for 10 passages.

2.1.2 Assay of parasite growth inhibition [2-4]

The values of growth inhibition of *L. donovani* promastigotes were determined based on the metabolism of resazurin to resorufin by aerobic respiration of metabolically active cells using 384-well plates that were seeded with *L. donovani* promastigotes ($5x10^4$ cells per well) and incubated with tested compounds for 3 days followed by addition of Resazurin sodium salt (200 lM; R7017; Sigma-Aldrich, St. Louis, MO, USA) and further incubation for 5 h then the cells were fixed (4% paraformaldehyde). The plates were analysed using a Victor3TM plate reader (PerkinElmer, Inc., Waltham, MA, USA) at 590 nm (emission) and 530 nm (excitation). Miltefosine and erufosine were used as the reference standards. All measured and calculated values are the averages of triplicates. The dose-response curves were generated by GraphPad Prism 6 software using a sigmoidal dose-response equation with a variable hill slope option.

2.2. *In vitro* safety evaluation [2-4]

PMA-treated THP-1 human monocytic cells (ATCC, USA) were seeded at $0.8x10^4$ cells per well in a 384-well culture plate (Greiner BioOne, Kremsmunster, Austria) in RPMI-1640 complete medium supplemented with 10% FBS. After 48 h of incubation at 37 °C in the presence of 5%

CO_2, infected THP-1 cells were treated with miltefosine (at 80 mM, positive control), and tested compounds (at

200 μM). The negative control consisted of THP-1 infected with the parasite with only 0.5% DMSO. After 72 h, the cells that were infected and treated with the drug were washed with serum-free RPMI-1640 medium. The cells and parasites were stained using 5 mM DAPI and 4% PFA. The images were acquired based on reading using an Operetta® automated microscope (PerkinElmer, Inc., Waltham, MA 02451, USA). They were further analysed using Columbus™ (PerkinElmer, Inc. Waltham, MA, USA) software to quantify parasite numbers, and host cell numbers. In brief, large-sized nucleus of host cells was first detected using DAPI signal and the host cell boundary masking was performed using the low-intensity signals from cytosols. Then the small-sized nucleus signal by DAPI was used to identify parasites within the area of the masked host cell. The DRC results were further assessed in a dose-dilution manner (two-fold serial dilution for 10 points starting from 200 μM).

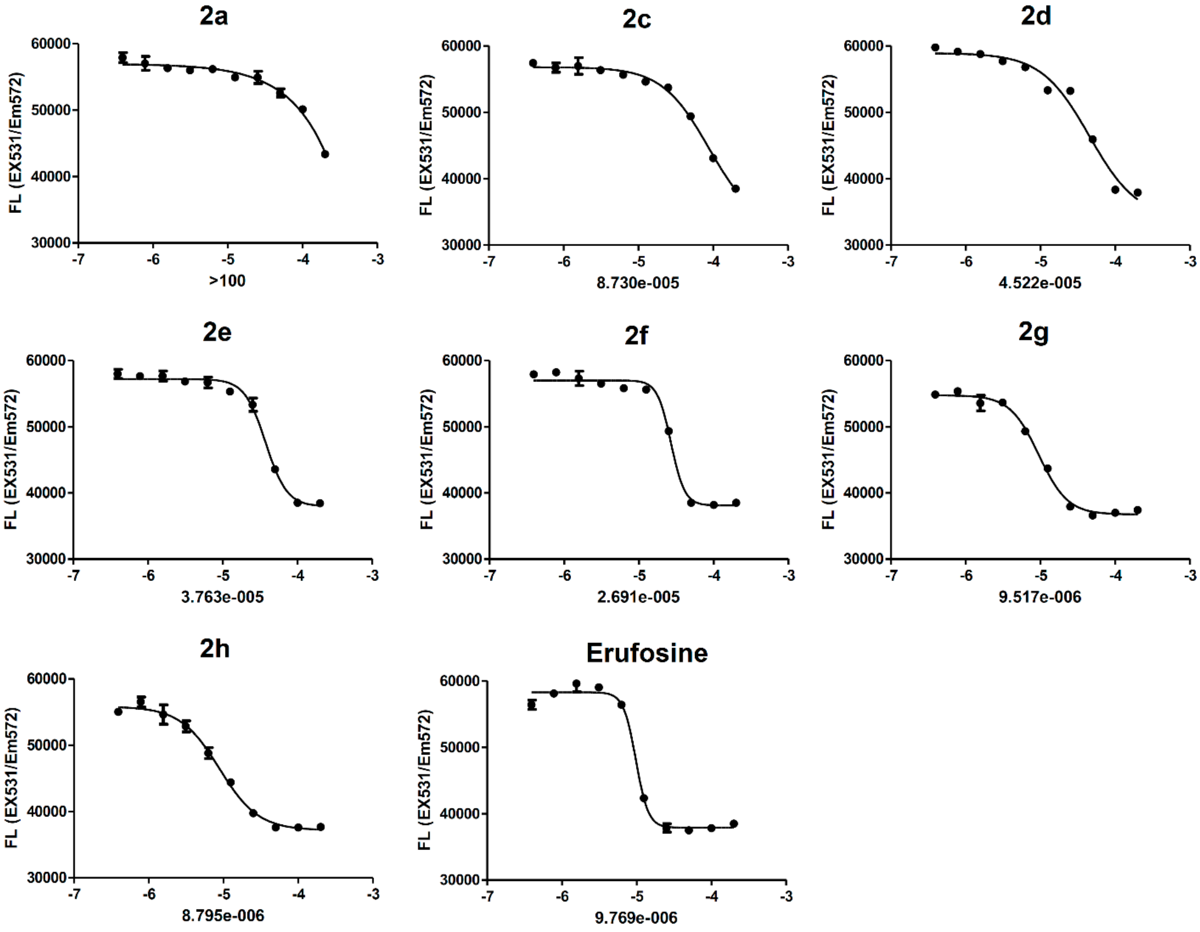

References:

[1] Y.J. Yoo, D.H. Nam, S.Y. Jung, J.W. Jang, H.J. Kim, C. Jin, A.N. Pae, Y.S. Lee, Synthesis of cinnamoyl ketoamides as hybrid structures of antioxidants and calpain inhibitors, Bioorg Med Chem Lett, 21 (2011) 2850-2854.

[2] A.H.E. Hassan, T.-N. Phan, Y. Choi, S. Moon, J.H. No, Y.S. Lee, Design, Rational Repurposing, Synthesis, In Vitro Evaluation, Homology Modeling and In Silico Study of Sulfuretin Analogs as Potential Antileishmanial Hit Compounds, Pharmaceuticals, 15 (2022) 1058.

[3] T.N. Phan, K.H. Baek, N. Lee, S.Y. Byun, D. Shum, J.H. No, In Vitro and in Vivo Activity of mTOR Kinase and PI3K Inhibitors Against Leishmania donovani and Trypanosoma brucei, Molecules, 25 (2020) 1980.

[4] A.H.E. Hassan, T.N. Phan, S. Yoon, C.J. Lee, H.R. Jeon, S.H. Kim, J.H. No, Y.S. Lee, Pyrrolidine-based 3-deoxysphingosylphosphorylcholine analogs as possible candidates against neglected tropical diseases (NTDs): identification of hit compounds towards development of potential treatment of Leishmania donovani, J Enzyme Inhib Med Chem, 36 (2021) 1922-1930.

NMR Spectra

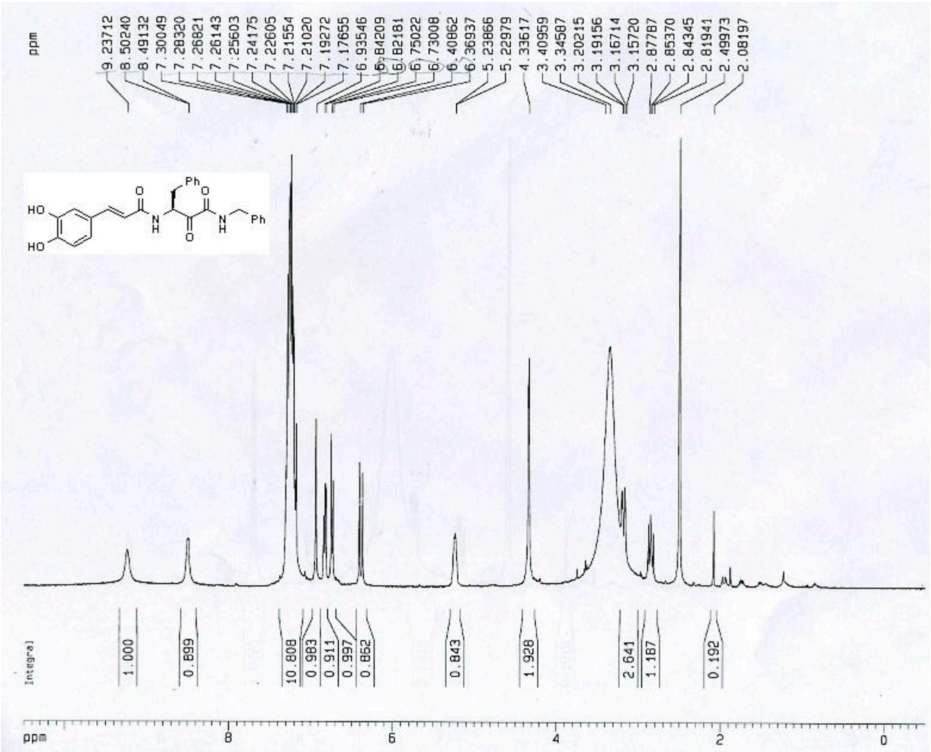

^{1}H-NMR Spectra of compound 2a

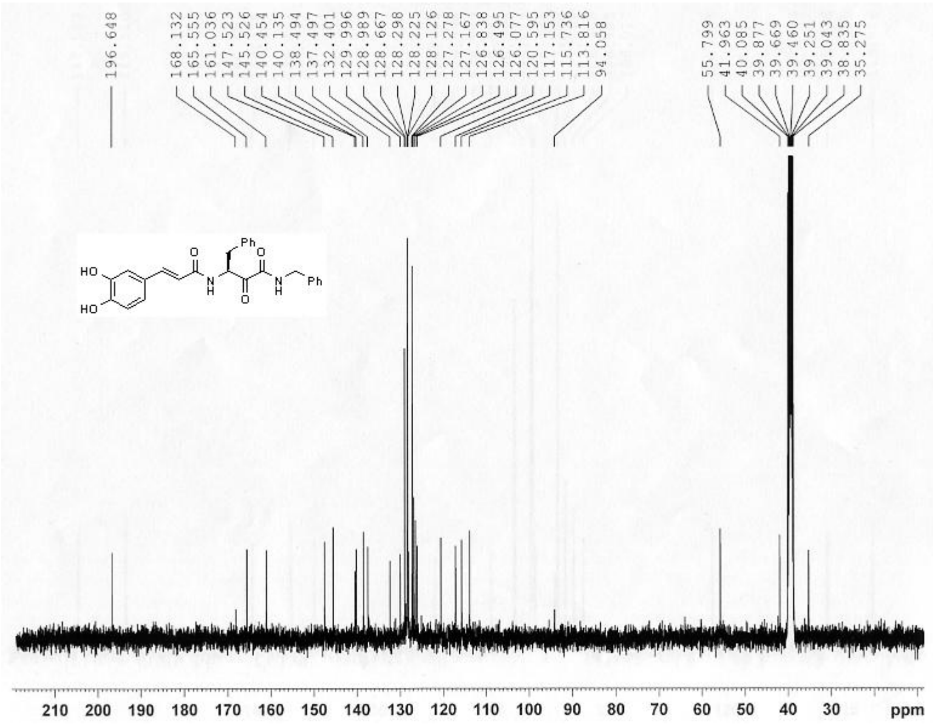

^{13}C-NMR Spectra of compound 2a

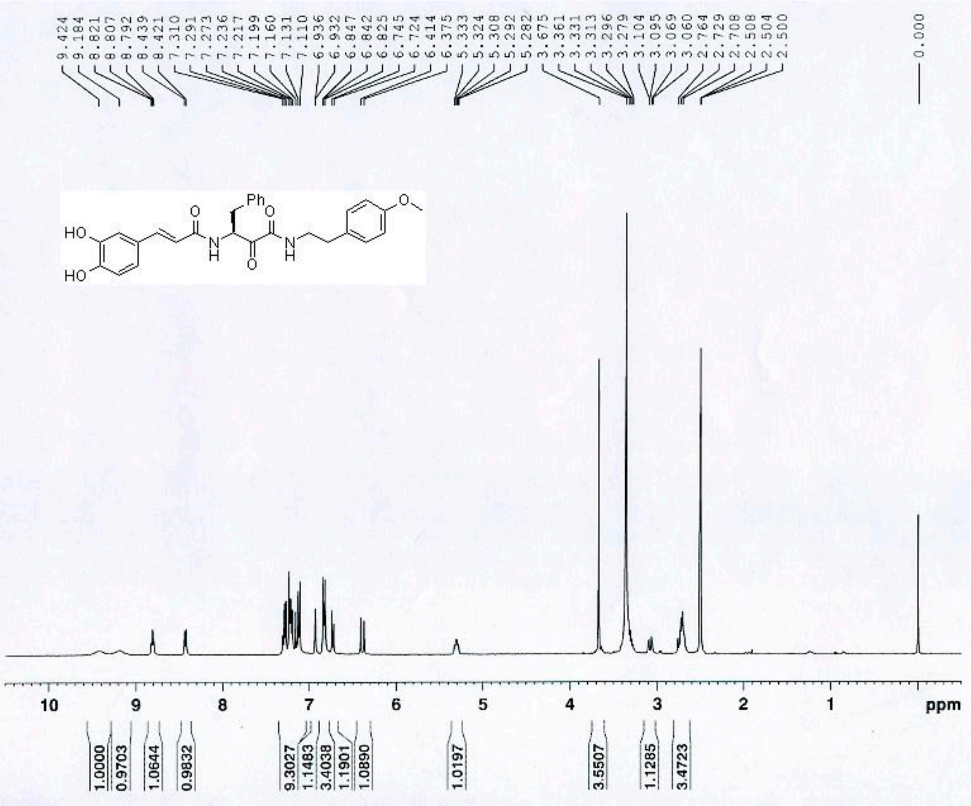

^{1}H-NMR Spectra of compound 2b

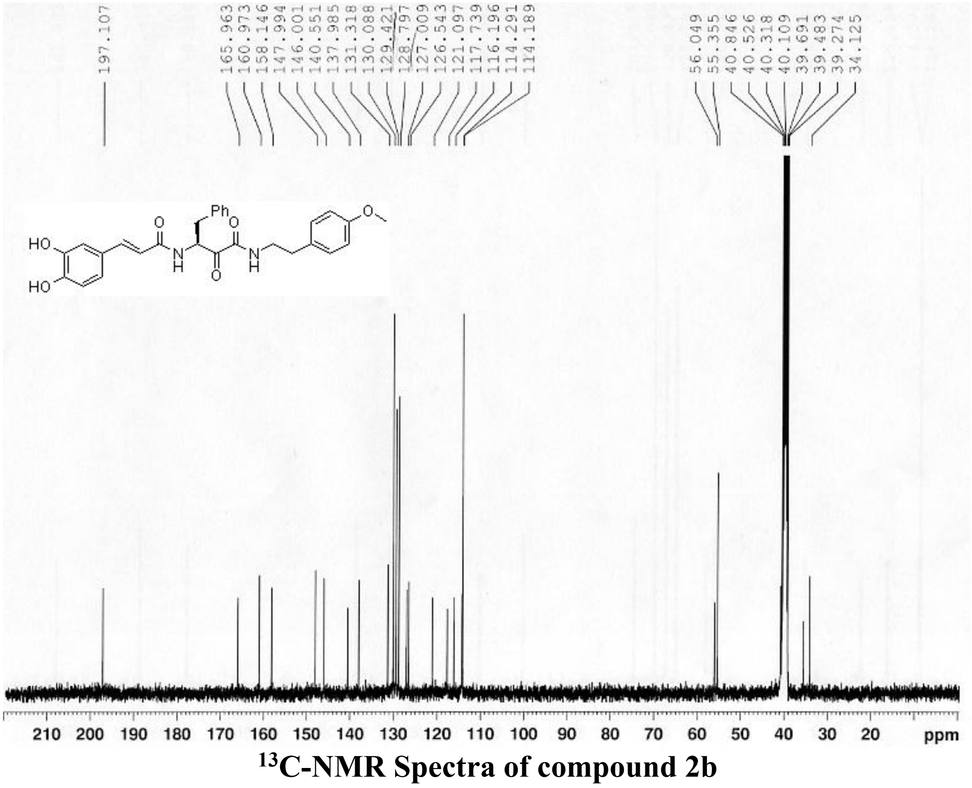

^{13}C-NMR Spectra of compound 2b

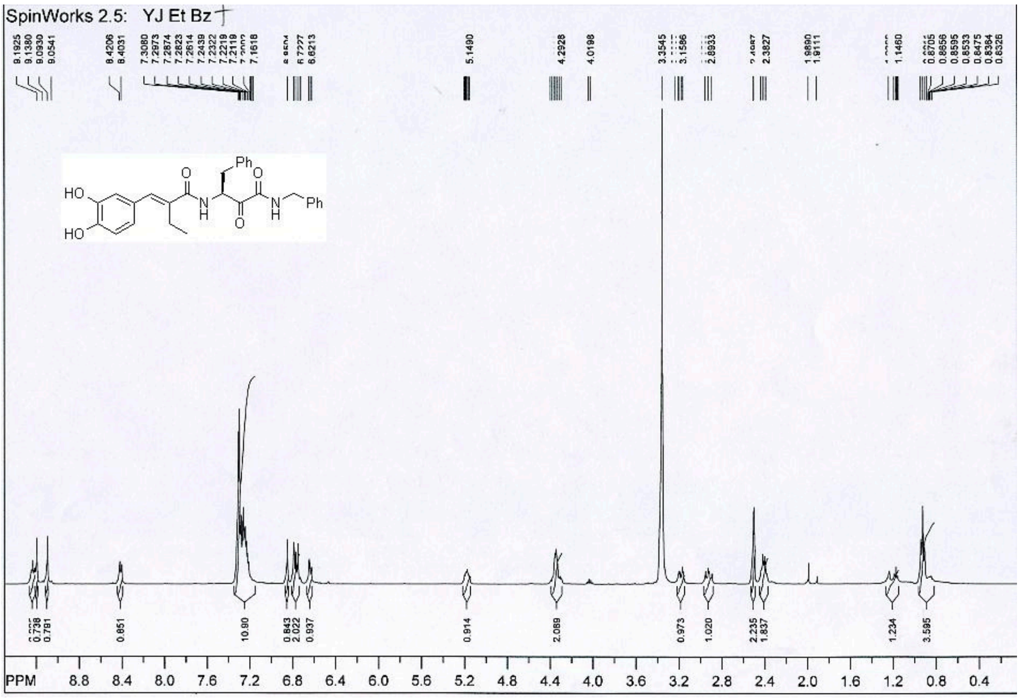

^{1}H-NMR Spectra of compound 2c

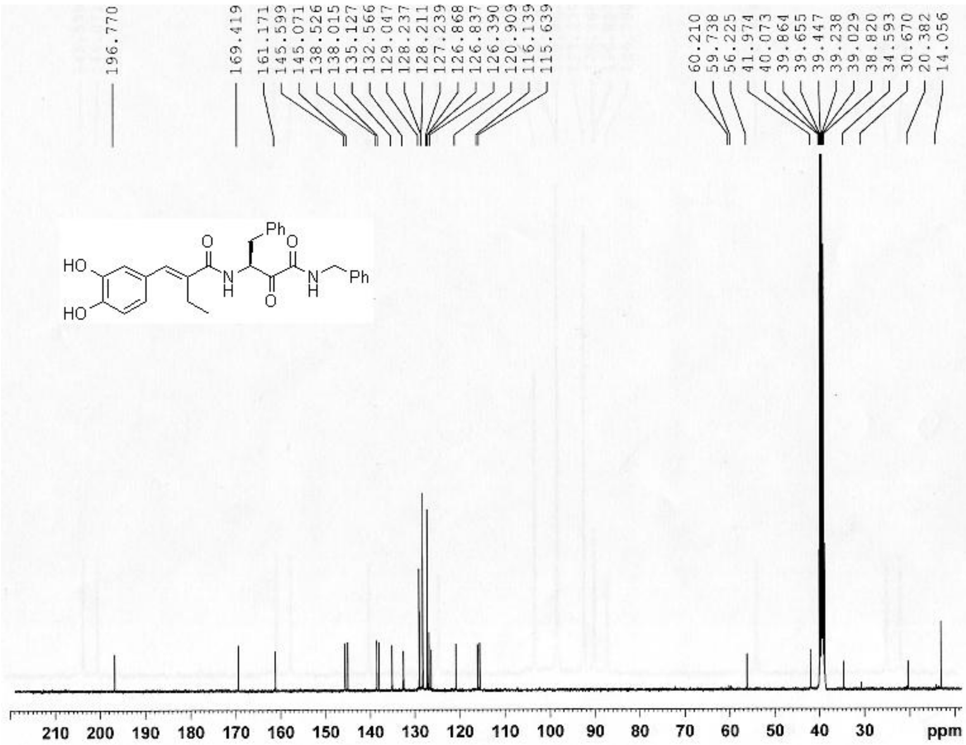

^{13}C-NMR Spectra of compound 2c

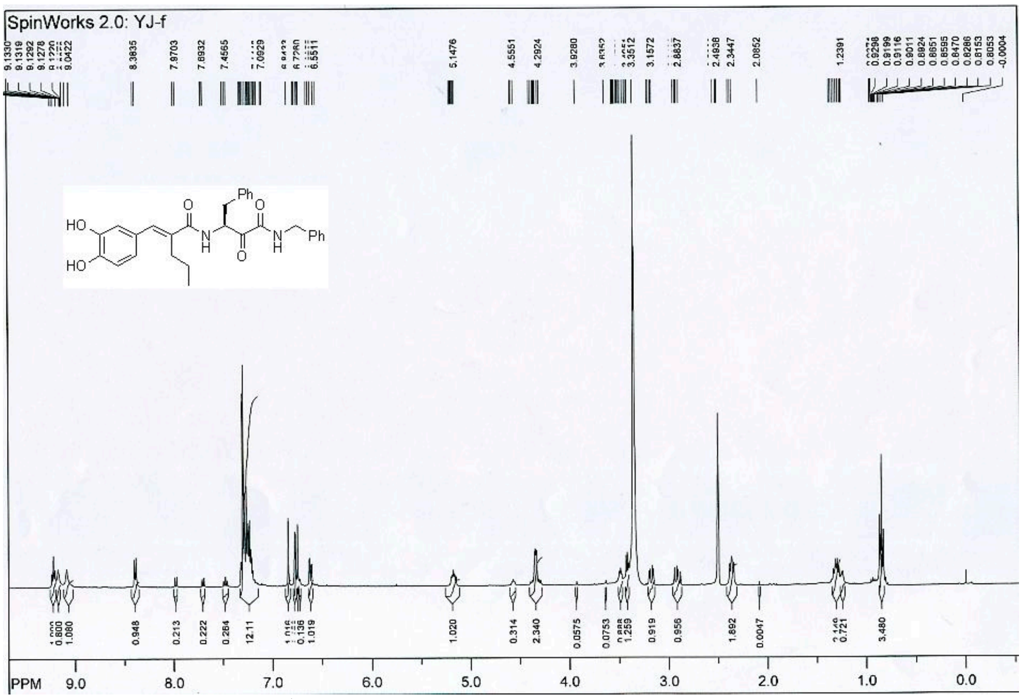

^{1}H-NMR Spectra of compound 2d

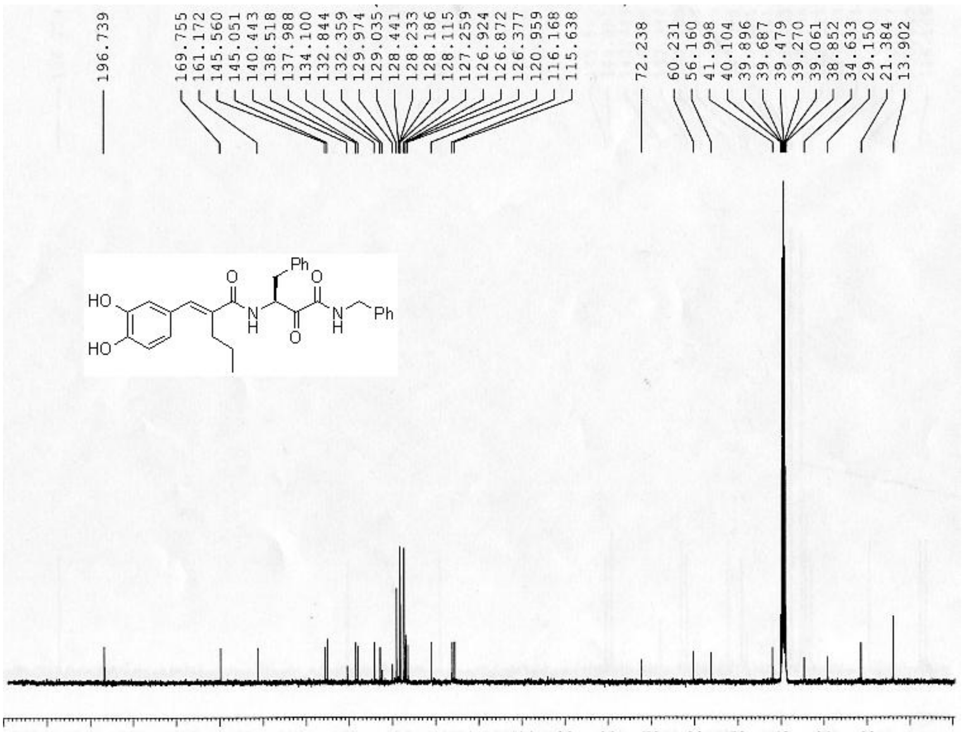

^{1}H-NMR Spectra of compound 2d

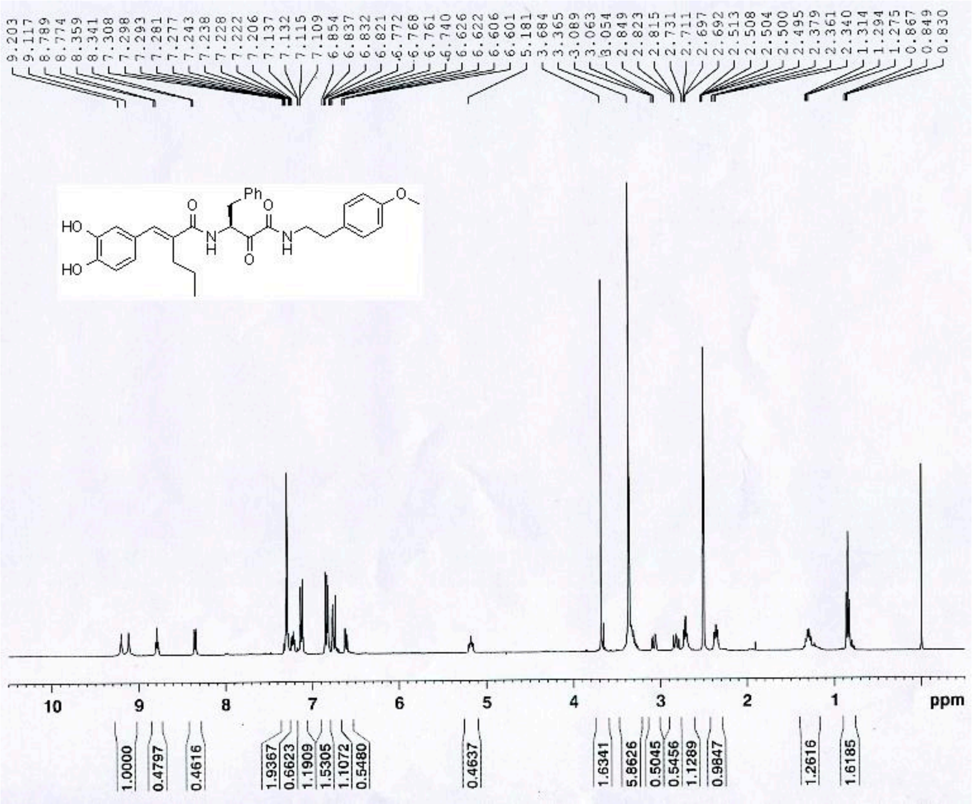

^{1}H-NMR Spectra of compound 2e

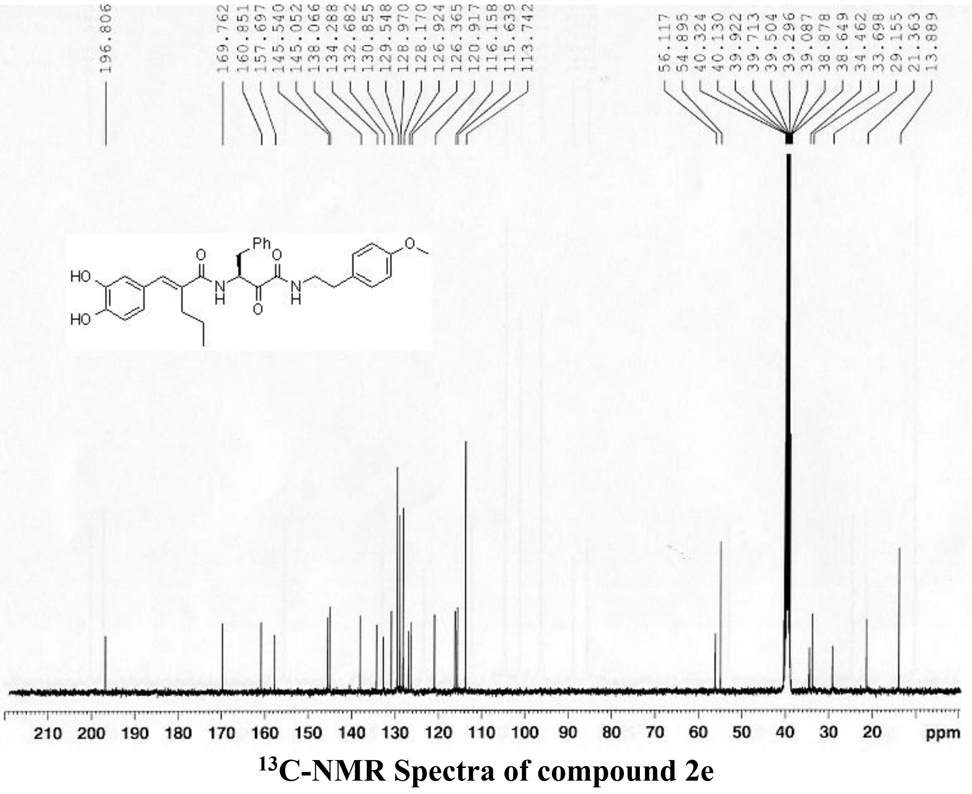

^{13}C-NMR Spectra of compound 2e

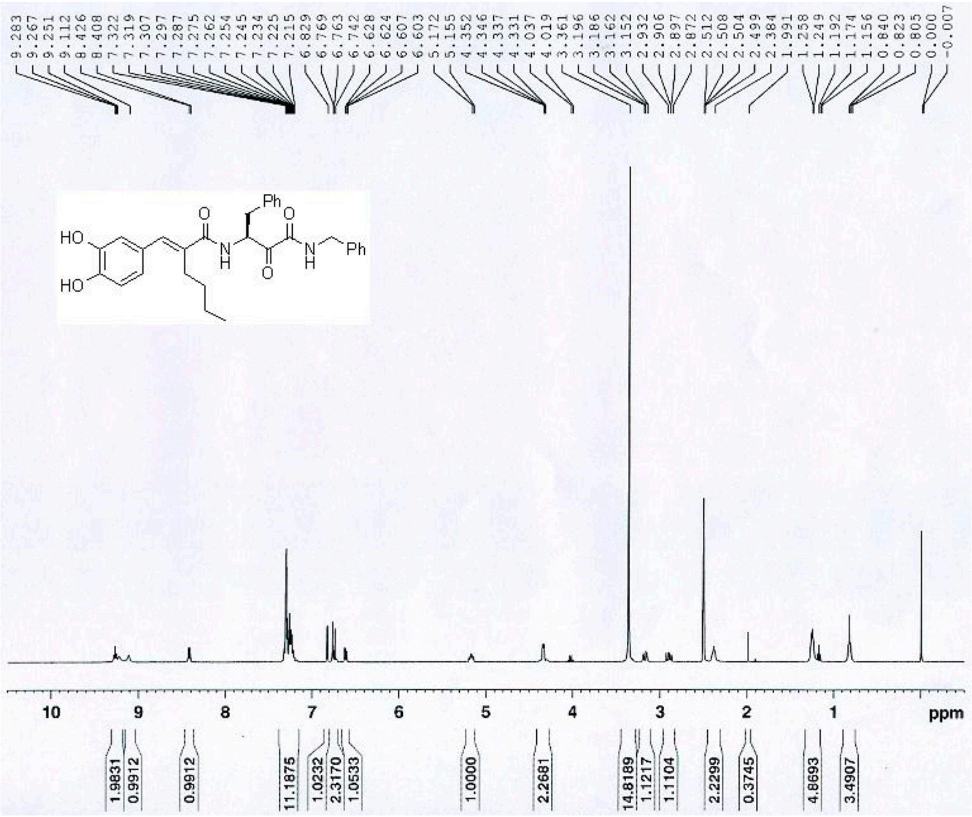

^{1}H-NMR Spectra of compound 2f

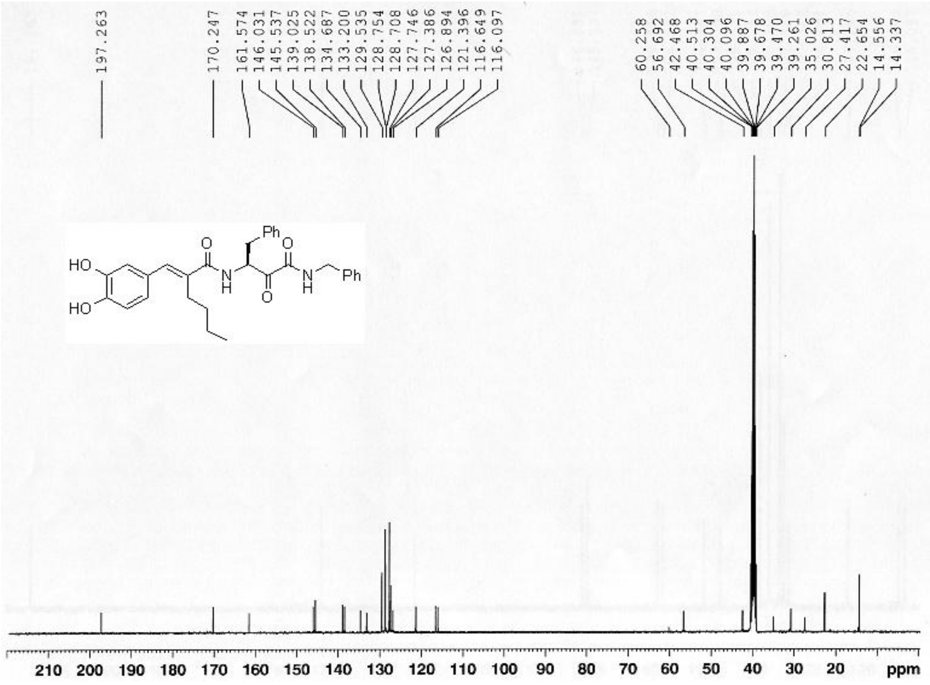

^{13}C-NMR Spectra of compound 2f

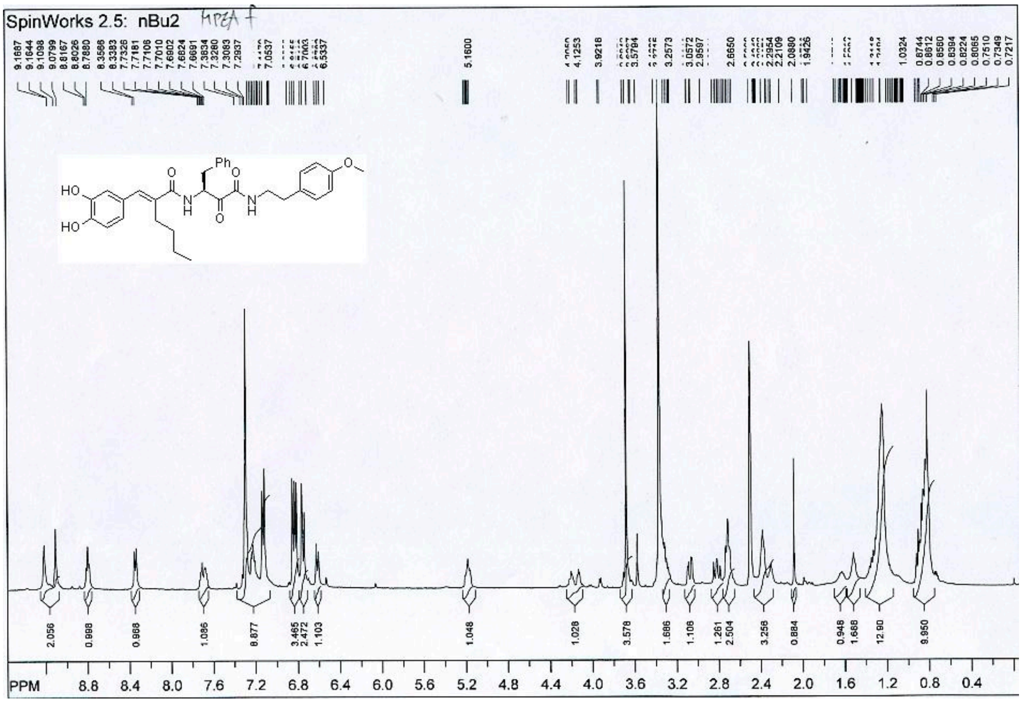

^{1}H-NMR Spectra of compound 2g

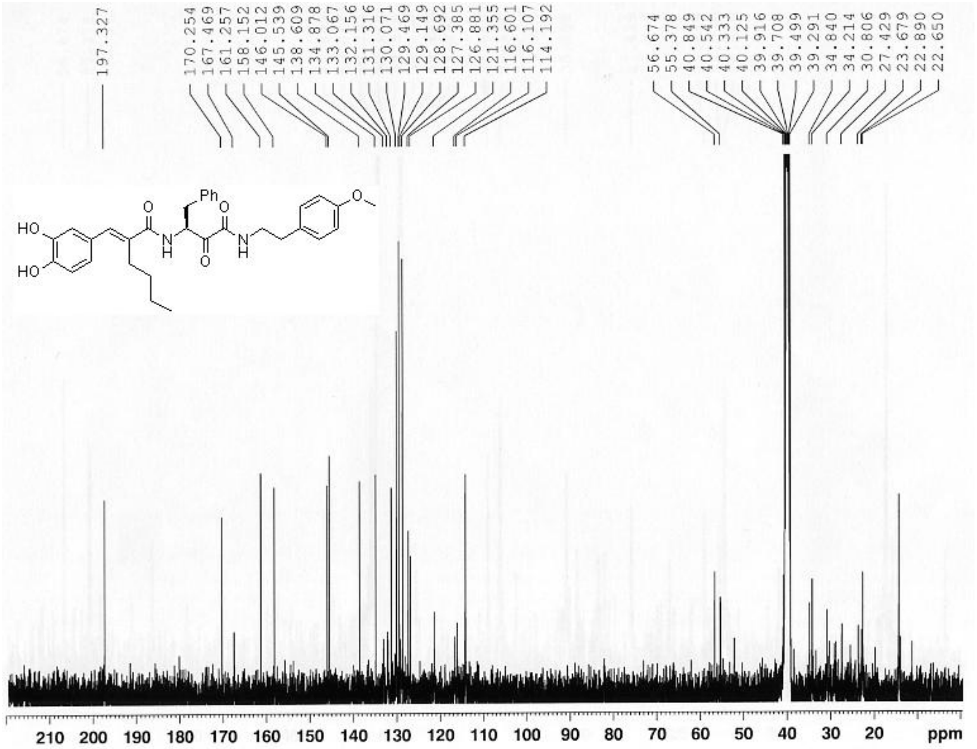

^{13}C-NMR Spectra of compound 2g

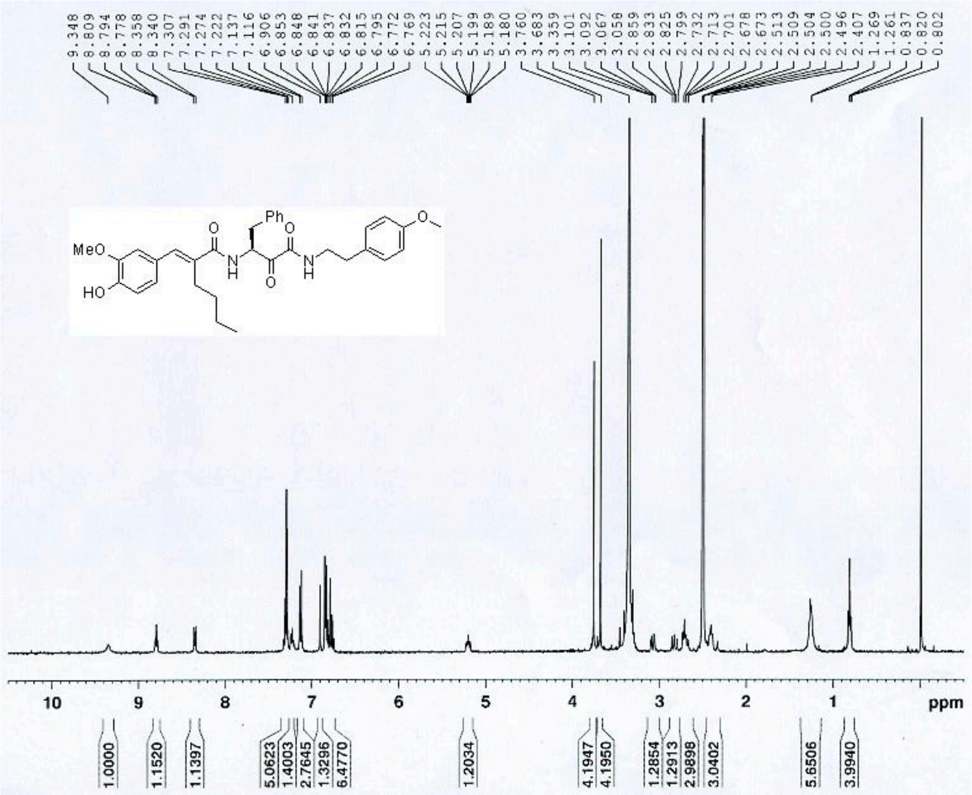

^{1}H-NMR Spectra of compound 2h

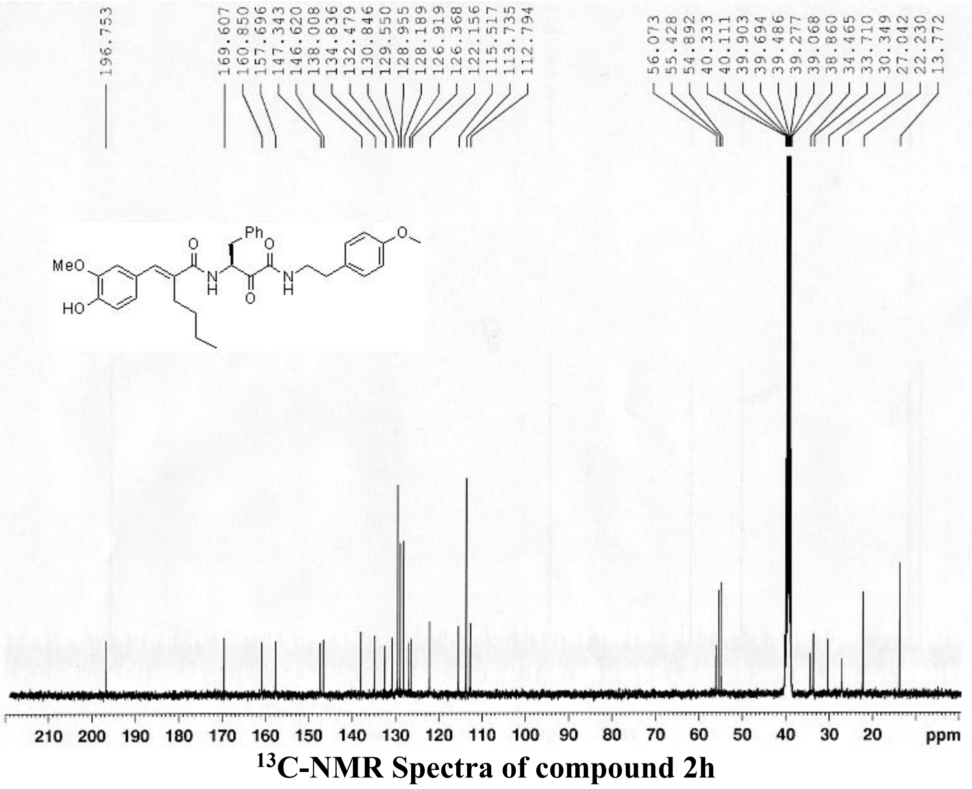

^{13}C-NMR Spectra of compound 2h

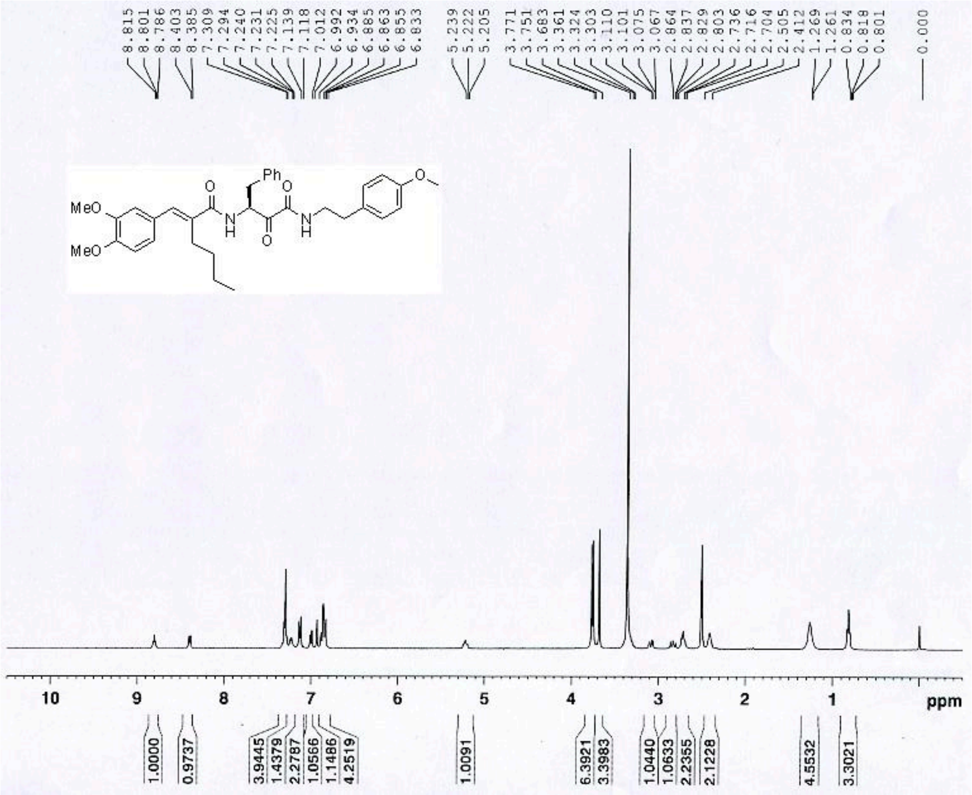

^{1}H-NMR Spectra of compound 2i

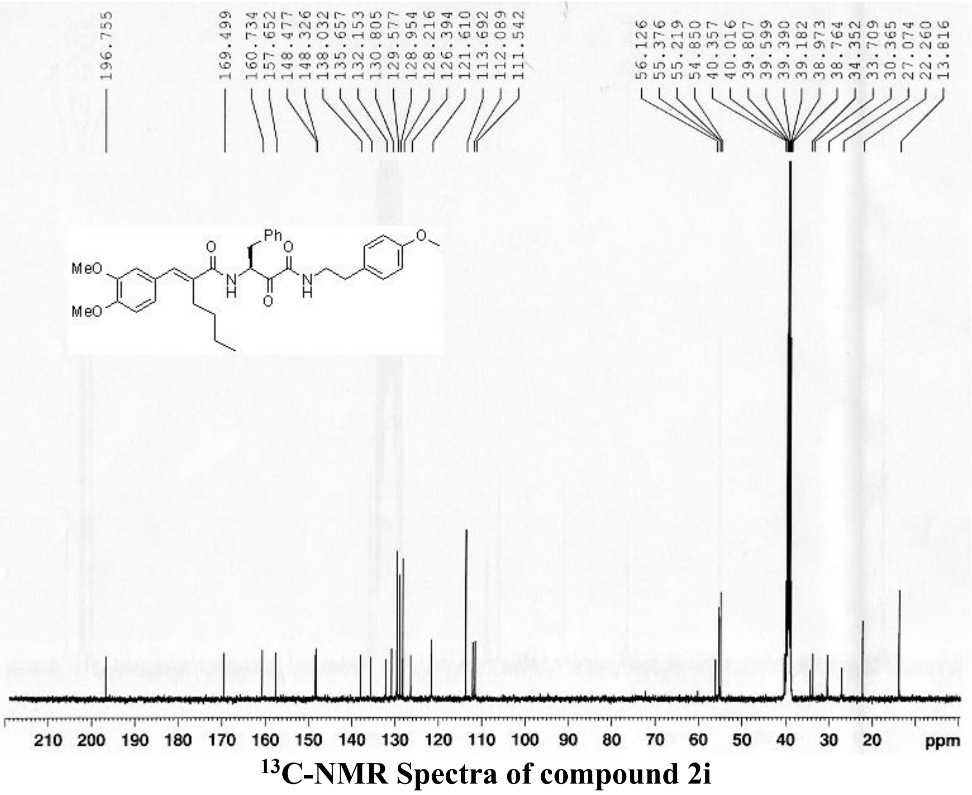

^{13}C-NMR Spectra of compound 2i
